# Effectiveness of brief alcohol interventions for pregnant women: a systematic literature review and meta-analysis

**DOI:** 10.1186/s12884-023-05344-8

**Published:** 2023-01-24

**Authors:** Svetlana Popova, Danijela Dozet, Ekta Pandya, Marcos Sanches, Krista Brower, Lidia Segura, Steven J. Ondersma

**Affiliations:** 1 Institute for Mental Health Policy Research, Centre for Addiction and Mental Health, 33 Ursula Franklin Street, Toronto, ON M5S 2S1 Canada; 2 Dalla Lana School of Public Health, University of Toronto, 155 College Street, Toronto, ON M5T 3M7 Canada; 3 Factor-Inwentash Faculty of Social Work, University of Toronto, 246 Bloor Street W, Toronto, ON M5S 1V4 Canada; 4 Institute of Medical Science, Faculty of Medicine, University of Toronto, Medical Sciences Building, 1 King’s College Circle, Toronto, ON M5S 1A8 Canada; 5 Biostatistics Core, Centre for Addiction and Mental Health, 33 Ursula Franklin Street, Toronto, ON M5S 2S1 Canada; 6 Edmonton Oliver Primary Care Network, 130, 11910-111 Avenue NW, Edmonton, Alberta Canada; 7 Department of Educational Research, Lancaster University, Lancaster, LA1 4YW UK; 8 Program on Substance Abuse, Public Health Agency of Catalonia, C.Roc Boronat 81 – 95, 08005 Barcelona, Catalonia Spain; 9 Department of Obstetrics, Gynecology, & Reproductive Biology, Michigan State University, 965 Wilson Rd, East Lansing, MI 48824 USA

**Keywords:** Alcohol, Brief interventions, Medical disorders in pregnancy, Substance misuse in pregnancy, Birth outcomes, Fetal alcohol spectrum disorder

## Abstract

**Background:**

Prenatal alcohol exposure (PAE) can result in a range of adverse neonatal outcomes, including Fetal Alcohol Spectrum Disorder (FASD). This systematic review and meta-analysis sought to investigate the effectiveness of brief interventions (BIs) in eliminating or reducing 1) alcohol consumption during pregnancy; and 2) PAE-related adverse neonatal outcomes; and 3) cost-effectiveness of BIs.

**Method:**

We conducted a systematic literature search for original controlled studies (randomized control trials (RCTs); quasi-experimental) in any setting, published from 1987 to 2021. The comparison group was no/minimal intervention, where a measure of alcohol consumption was reported. Studies were critically appraised using the Centre for Evidence-based Medicine Oxford critical appraisal tool for RCTs (1). The certainty in the evidence for each outcome was assessed using the GRADE (Grading of Recommendations Assessment, Development and Evaluation) (2). Meta-analysis of continuous and binary estimates of effect-size for similar outcome measures for BIs versus control groups were pooled and reported as mean difference (MD) Hedges’ g and odds ratios (ORs), respectively.

**Results:**

In total, 26 studies, all from high income countries, met inclusion criteria. Alcohol abstinence outcome available in 12 studies (*n* = 2620) found modest effects in favor of BIs conditions by increasing the odds of abstinence by 56% (OR = 1.56, 95% confidence interval (CI) = 1.15–2.13, I^2^ = 46.75%; *p* = 0.04). BIs effects for reduction in mean drinks/week (Cohen’s d = − 0.21, 95%CI = - 0.78 to 0.36; *p* = 0.08) and AUDIT scores (g = 0.10, 95%CI = − 0.06 to 0.26; *p* = 0.17) were not statistically significant. Among seven studies (*n* = 740) reporting neonatal outcomes, BI receipt was associated with a modest and significant reduction in preterm birth (OR = 0.67, 95% CI = 0.46–0.98, I^2^ = 0.00%; *p* = 0.58). No statistically significant differences were observed for mean birthweight or lower likelihood of low birth weight (LBW). Certainty in the evidence was rated as ‘low’. No eligible studies were found on cost-effectiveness of BIs.

**Conclusion:**

BIs are moderately effective in increasing abstinence during pregnancy and preventing preterm birth. More studies on the effectiveness of BIs are needed from low- and middle-income countries, as well as with younger mothers and with a broader range of ethnic groups.

**Supplementary Information:**

The online version contains supplementary material available at 10.1186/s12884-023-05344-8.

## Background

Alcohol use during pregnancy is a significant health concern globally. Decades of research have provided overwhelming evidence that alcohol is a teratogen that can significantly harm the developing fetus. Prenatal alcohol exposure (PAE) increases the risk for many adverse maternal and neonatal outcomes, including spontaneous abortion [1], stillbirth [2], low birthweight (LBW) [3, 4], intrauterine growth restriction (IUGR) [3, 5], and preterm birth [6, 7]. PAE can also result in Fetal Alcohol Spectrum Disorder (FASD) in the child, a lifelong neurodevelopmental disorder that poses significant physical, mental and social challenges to affected individuals. Even relatively low levels of maternal alcohol consumption can cause FASD in the child [8]. FASD affects approximately one in every 13 children who were prenatally-alcohol exposed [9], though this disorder is widely misdiagnosed and underdiagnosed [10]. FASD can lead to many organ or system defects and is associated with more than 400 disease conditions [11]. This poses an enormous cost to service systems related to increased use of health care services, involvement in child welfare, and correctional systems [12, 13].

Globally, approximately 10% of women consume alcohol during pregnancy and 3% of these women report having 4 or more drinks in one sitting (i.e., binge drinking) [14]. These prevalences are expected to increase based on global trends such as increasing alcohol consumption among women of childbearing age, increasing social acceptability of women’s alcohol use, as well as recent changes in alcohol use patterns due to the COVID-19 pandemic, all of which will increase the number of alcohol-exposed pregnancies and increase the risk of FASD [15–17]. Alcohol use during pregnancy may be more common among women who have been exposed to intimate partner violence, have limited access to education or prenatal care, have substance use disorders, or use tobacco [18]. In particular, negative attitudes toward the pregnancy or attitudes conducive of alcohol use during pregnancy are both predictive of maternal alcohol consumption [19, 20]. Stigma experienced by pregnant women and by mothers of children with FASD can lead to these women avoiding contact with services that could help them [21]. Notably, any decrease in alcohol use during pregnancy is beneficial in terms of fetal health outcomes [22], suggesting a potentially powerful role for obstetricians and midwives in preventing alcohol-related harms during pregnancy.

Prevention and treatment of substance use in pregnancy is central to the 2015 United Nations Sustainable Development Goals [23], and the WHO recommendations for FASD prevention are based on universal screening and early intervention for PAE [24, 25]. Brief interventions (BIs) are an evidence-based, healthcare-centric approach consisting of a short advice or counselling session wherein a healthcare provider seeks to promote behavioral change, typically using motivational techniques. BIs are typically paired with universal proactive screening in approaches referred to as Screening and Brief Intervention (SBI) or Screening, Brief Intervention, and Referral to Treatment (SBIRT). In obstetric settings, BIs present the opportunity to educate and empower women to make their own choices to promote healthy outcomes for themselves and their children. BIs may be a low-cost option to prevent PAE that could simultaneously strengthen the provider-patient relationship and reduce the likelihood of FASD in the child. The efficacy of person- and technology-delivered BIs has been studied extensively in general populations [26], however, fewer studies have examined their utility during pregnancy [27]. Although studies in this area have accumulated sufficiently to support early meta-analysis [28], this systematic review and meta-analysis sought to update those efforts with more recent studies and to add analysis of BI effects on neonatal outcomes.

### Objectives

This study aimed to investigate the effectiveness of alcohol brief interventions (BIs) in eliminating or reducing 1) alcohol consumption during pregnancy and 2) PAE-related adverse neonatal outcomes; and to investigate the economic evaluation of BIs during pregnancy.

## Method

Two methods were employed: 1) a comprehensive systematic literature review; and 2) a meta-analysis (protocol not registered). A comprehensive systematic literature search was conducted for original quantitative studies (randomized control trials (RCTs); quasi-experimental) that reported on the effectiveness of alcohol BIs in pregnant women in any setting and /or PAE-related adverse neonatal outcomes. The search focused on studies published from 1987 to 2021, and the search was not restricted geographically or by language. Online databases: MEDLINE Ovid (All), CINAHL, PsycINFO, and EMBASE, Cochrane Central Register of Controlled Trials (CENTRAL) were searched. Web of Science (*Social Citation Index Expanded, Social Sciences Citation Index, Science/ Social Science and Humanities Conference Proceedings Citation Index, Emerging Sources Citation Index*), Google Scholar, International Committee on Harmonization of Good Clinical Practice (ICH-GCP) Clinical Trial Registry, European Monitoring Centre for Drugs and Drug Addiction, Canadian Centre on Substance Use and Addiction were also searched. A detailed literature search strategy (Additional File 1) and PRISMA Checklist (Additional File 2) are available.

Studies were included if they were experimental (individual or cluster-randomised control trials), or quasi-experimental (e.g., interrupted time series), included a control group (no care, or any routine treatment as usual), where the intervention was a BI, which was mentioned as brief/ short, and this was regardless of the duration, frequency of sessions, components, provided by a personnel or computer; were conducted with pregnant women; and alcohol was reported separately from other substance use (tobacco or drugs). We included studies regardless of maternal age, baseline alcohol use, parity, gestational age, or level of alcohol consumption during pregnancy. Studies were excluded if the BI was combined with pharmacological interventions on PAE or neonatal outcomes, or if the BI was conducted outside of the pregnancy period (e.g., preconception, post-partum, or breast-feeding).

### Article screening and data extraction

Study selection was conducted in two phases: 1) title and abstract screening; and 2) full-text screening. Screening at both phases was conducted independently by two investigators (EP and DD). Studies deemed to be potentially relevant that were published in languages other than English were translated either by colleagues fluent in the respective language or using Google Translate, and were subsequently cross-checked by a native speaker. Based on the articles agreed upon for inclusion, data were extracted and recorded in the Excel spreadsheet, designed based on Cochrane guidelines by one investigator and then independently cross-checked by a second investigator [29]. All discrepancies were reconciled by team discussion.

### Outcomes

The primary outcome for this study was change in alcohol use (quantity and frequency), comparing the BI group to the control group. Consumption of alcohol was most often reported as self-reports or other reports of drinking quantity (e.g., drinks per day/week), binge drinking frequency (e.g., number of binges per week), drinking frequency (e.g., drinking days per week), or alcohol abstinence. Reports of alcohol consumption may be captured by validated alcohol use screening tools for pregnant women, though this was not required for a study to be eligible. Odds ratios for alcohol abstinence were obtained by extracting frequency data from included studies or relevant information necessary to perform calculations.

The secondary measures of interest were neonatal outcomes related to PAE, including: FASD percentage/odds/risk; Appearance, Pulse, Grimace, Activity, and Respiration (APGAR; 1- or 5-minute) scores; small for gestational age (SGA); percentage of neonatal intensive care unit (NICU) admissions; mean body length; percentage of preterm birth; mean birth weight; and mean head circumference. These were obtained by extracting frequency or relevant information necessary to perform calculations, and comparing neonates whose mothers received a BI versus control group neonates.

The third outcome of the study was cost-effectiveness of BI versus controls (i.e., routine standard of care).

### Quality assurance

The quality of each RCT study was appraised using a tool specifically for use in the systematic evaluation of RCTs, developed by the Centre for Evidence Based Medicine (CEBM) from Oxford University [30]. Cluster-randomized control trials (C-RCTs) were assessed using a C-RCT-specific Cochrane tool [31] and pre-post studies without a control group were appraised using a National Institutes of Health (NIH) quality assessment tool [32]. The CEBM tool allowed to appraise each intervention study using specified criteria to assess the following domains: internal validity, randomization, measurement, reporting of results and external validity. Two investigators (EP and DD) independently conducted critical appraisal of the included studies and any disagreements within domains were reconciled using group discussion. To assess the certainty of  the overall evidence for each outcome, the GRADE (Grading of Recommendations Assessment, Development and Evaluation) approach [33] was used to examine studies pertaining to each outcome with respect to risk of bias, inconsistency, indirectness of evidence, imprecision, and publication bias.

### Meta-analysis

Meta-analyses of randomized and non-randomized trials [34, 35] were conducted using STATA 16 for alcohol consumption or neonatal outcomes that had at least two studies/ intervention arms and all the required statistical information to compute the pooled OR, or Cohen’s d or Hedge’s g for BIs versus control groups. A random-effects inverse-variance model was used under the assumptions that outcomes measures of studies are different yet related and follow normal distribution [36]. Random-effects models were used to conduct the analysis due to a high degree in heterogeneity across the studies, including variances in sampling, bias, study design, data collection measures and alcohol/neonatal outcomes [37].

The effect-size for continuous outcome measure of reduced alcohol consumption or neonatal outcomes (e.g., mean drinking days/week, binge drinking days, mean head circumference, mean birth weight, mean AUDIT scores) were presented as both unstandardized and standardized mean difference [38]. Binary outcomes for alcohol reduction and neonatal outcomes (e.g., proportion or risk ratio of alcohol abstainers, risky drinkers, binge drinkers, pre-term births, small for gestational age) were reported as ORs. The standardized mean differences were all reported as Hedge’s g to address the biased estimates of the population effect size, particularly for sample size < 20 [39]. A separate analysis was conducted to deal with outliers or influences (small study effect) by excluding studies whose 95% confidence intervals (95% CI) does not overlap with the pooled 95% CI and conducting influence analysis [36].

The extent of heterogeneity between the studies was quantified by calculating I^2^ statistic (0 to 40%: might not be important; 30 to 60%: moderate heterogeneity; 50 to 90%: substantial heterogeneity; 75 to 100%: considerable heterogeneity) [40]. In cases where studies had more than one follow-up/ repeated measures of alcohol use during pregnancy, the time-point selection was based on the author’s rationale for the importance of the time-point in the study (e.g., if there are three observation points in a study and 1 month, 2 month and 3 month and author suggests that alcohol use rate suddenly decreases in the second month and then stabilises after the third month post-intervention then the observation at 3 month should be considered).

### Unit-of-analysis

For cluster-randomized control trials where design effect/ multilevel analysis was not considered (inappropriate analysis), the external intra-class correlation coefficients (ICC) were adopted for similar clusters or outcomes to calculate effect size estimates and their standard errors/ deviations [41]. These results were then combined with individual randomised control trials (where individuals are both unit of randomisation and analysis) in the same meta-analysis for a pooled effect size. Each intervention arm was considered a unit of analysis in studies where more than one intervention arm was compared to the control group.

### Split control group analysis

A split-shared control group analysis was conducted to address the overweighing of studies where more than one intervention versus control arm were included in the meta-analysis. Each pair-wise comparison (arm) was included separately in the meta-analysis, wherein the shared control groups were almost evenly divided for the total number of controls and total of events observed in the control group in both arms [42].

### Subgroup analysis and meta-regression

To explore the moderate to considerate heterogeneity (I^2^ = 50–100%) and provide more conservative estimates of effect size, the influence of potential moderators on the effect size were studied [43]. Sub-group analyses would explore the influence of categorical moderators (e.g., study designs, age groups [44], and components of BI versus control groups [45] and meta-regression would explore the influence of continuous moderators; (e.g., baseline average alcohol consumption (average weekly), and baseline binge drinking days) [43, 46, 47]. These explanatory variables were selected as potential moderators based on existing studies of alcohol use interventions in pregnant and non-pregnant populations [45, 48]. The subgroup analysis or meta-regression would be conducted when all of the following conditions were met: a) the combined pooled effect-estimate has moderate to considerate heterogeneity; b) have at least 3 studies in each group for subgroup analysis [49] and a minimum of 6 studies for meta-regression [50], and c) information on these factors are available and comparable (measured consistently across the studies) [51].

### Sensitivity analysis

To explore the influence of including non-randomized studies in meta-analysis [34], it is necessary to conduct sensitivity analysis to obtain the effect size estimates after removing non-randomized studies from the meta-analysis. Sensitivity analysis would be conducted separately for alcohol use outcomes and neonatal outcomes.

### Risk of bias/publication bias

The publication bias or small study effect assessment was conducted using the funnel plot of standard error plotted against the effect-size, Peters test and Egger’s weighted regression test [52]. At least 10 studies were required in the meta-analysis in order to have enough power to distinguish real asymmetry or skewed distribution in the funnel plots [53]**.** The *p*-value of < 0.05 in Egger’s weighted regression test suggests significant publication bias or small study-effect [52].

## Results

**Fig. 1:**
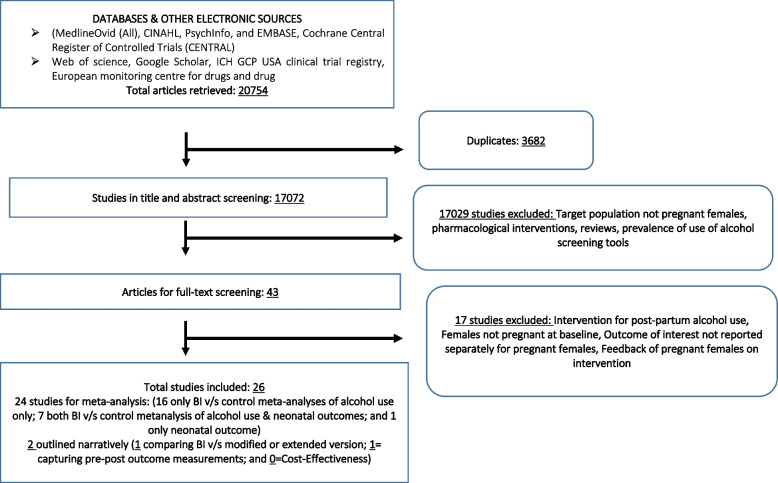
Study selection flow diagram

The systematic literature search generated 20,754 studies in total, identified from electronic sources and hand-searching. A total of 26 articles met the inclusion criteria and were included in the review (Additional Files 3 and 4). Of these studies, 25 had BI and control groups, and 24 studies were eligible for inclusion in the meta-analysis: 17 studies reported alcohol use only, 6 studies reported both alcohol use and neonatal outcomes, and 1 study reported neonatal outcomes only. Out of 23 studies with alcohol use outcomes, a total of 13 had similar outcomes that were included in the meta-analysis and used to obtain pooled estimates. Out of 7 studies with neonatal outcomes, a total of 5 had similar outcomes that were included in the meta-analysis to obtain pooled estimates. The remaining two studies reported alcohol use (one study without a control group, and one study in which the control group received treatment similar to the BI group) and were synthesized narratively. No eligible studies were found on cost-effectiveness of BIs (Fig. 1).

Most of the studies (*n* = 16; 61.5%) were conducted in the USA [54–69], followed by two studies (7.7%) in South Africa [70, 71], and one study (3.8%) each in Brazil [72], Ireland [73], Israel [74], Netherlands [75], Norway [76], Spain [77], Sweden [78], and UK [79].

### Quality assessment

All 26 studies were critically appraised: 21 studies were RCTs and were assessed using the CEBM tool (Additional File 5); 3 studies were cluster-randomized control trials (C-RCTs) and assessed using a C-RCT-specific Cochrane tool [31]; and one study was a pre-post study without a control group, appraised using an NIH quality assessment tool [32] (Additional File 6). Individuals were not randomized in 3 (12%) studies [59, 73, 76, 78], randomization was unclear in one (3.8%) study [79] and 3 (11.5%) studies were cluster-randomized control trials (C-RCTs) [54, 71, 75]. In most cases, when assignment of treatment condition was done at the individual level and this was not randomized, the condition was assigned based on study site. Baseline characteristics (e.g., demographics; baseline alcohol use levels) were not comparable or were unclear for intervention versus control in 11 (44%) studies [54, 55, 57, 59, 64, 65, 71–75], which could not be accounted for in the analysis. In four (16%) studies, the intervention and control groups were treated equally, apart from the treatment itself [54, 55, 61, 62]. Participants were lost to follow-up and/or not analyzed in the group to which they were assigned in nine studies (38.5%) [54, 61–63, 74–76, 78, 79]. In all, 11 studies (44%) did not explicitly mention conducting intention to treat (ITT) analysis [55, 58, 61, 62, 66, 67, 72, 74, 76, 78, 79]. In total, 18 studies (88.5%) did not mention blinding the assessor (data analyst) or the follow-up researcher to the intervention condition [56–60, 63–65, 67, 69, 71–76, 78, 79]. Two (8%) studies did not report the effect-size [65, 76]. In total, 6 (24%) studies did not report important statistics (e.g., range; 95% CI; *p*-value, etc.) to estimate the true effect [59, 61, 65, 66, 76, 79], (Additional File 4). The certainty in evidence for each outcome included in the meta-analysis (6 outcomes) was assessed using the GRADE approach for 1) all study designs; and 2) RCTs only, for each outcome (See Additional File 6). Based on these assessments, the certainty in evidence for all meta-analyzed outcomes was “low”, with RCT groupings baring no importance on the overall rankings.

### Study settings and designs

Half of the studies (*n* = 13; 50%) were conducted in obstetrics or prenatal clinics within hospitals [54, 55, 57, 61, 62, 64–69, 77, 79], followed by 8 (30.8%) in clinics or health care centers in rural or urban areas [56, 58–60, 63, 70, 71, 73], and 2 (7.8%) in midwives’ offices [75, 76], and maternity care or women health centers [72, 78]. One (3.8%) study was conducted in an in-patient pre-delivery and emergency unit of the hospital [74] (Additional File 3).

Among the 25 studies that had a control group, 18 (72%) studies were RCTs [55–58, 60–69, 72–74, 77], and 3 (12%) were C-RCTs [54, 71, 75]. Among the remaining four studies, two studies selected non-equivalent controls from the same setting using different time points [76, 78], and two studies examined controls from a different setting [59, 79] (Additional File 3).

### Characteristics of pregnant women included in the studies

In total, 11 (42.3%) studies had age criteria for inclusion of pregnant women in the study, with the majority (*n* = 9 studies, 34.6%) including only pregnant women aged 18 years and above [56, 60, 64–66, 68, 72, 73, 75]. One (3.8%) study included pregnant women 16 years and over [57], and one (3.8%) study included participants 15 years and over [71].

In total, 14 studies recruited pregnant women based on their gestational age at baseline: majority of the studies recruited in third trimester (n = 9, 34.6%) [55–58, 60, 61, 64, 65, 74], three studies (11.5%) in second trimester [67, 68, 71], and one study each that included pregnant women in their first trimester [75] and between 20 and 30 weeks of pregnancy, respectively [72].

The majority of studies (*n* = 15; 57.7%) recruited pregnant women who had indicated some alcohol use (any level) during their pregnancy [54, 56, 58, 61–63, 65, 66, 71, 73, 75–79], followed by 6 studies (23.1%) where women indicated alcohol or other substance use (any level of drinking, reported separately from other substances) [57, 59, 60, 67, 72, 74], four studies (15.4%) that specifically included women who were deemed risky-level drinkers at baseline [55, 64, 68, 70], and one study (3.8%) that included moderate-level drinkers [69].

### Screening tools used at baseline and post-intervention

More than one-third of the included studies (*n* = 9, 34.6%) used T-ACE (*positive/ scores ≥ 2*) [55, 58, 61, 64, 66, 67, 72, 75], followed by timeline follow back (TLFB) used in seven (26.9%) studies [55, 57, 64–66, 69, 77], and six (23.1%) studies that used the Alcohol Use Disorders Identification Test (AUDIT-10) [56, 65, 70, 71, 74, 77] for screening alcohol use among pregnant women (Additional File 3).

### Components of intervention and control groups

The intervention group received alcohol use screening (T-ACE, AUDIT-10 or TLFB) in all studies. In addition to alcohol use screening, 15 (57.7%) studies also provided Motivational Interviewing (MI) [56–58, 61, 62, 64–67, 70, 71, 74, 75, 77, 78], 3 (11.5%) studies used Motivational Enhancement Therapy (MET) and Cognitive Behavioural Therapy (CBT) combined (MET-CBT) [55, 57, 72], and 2 studies used MET alone [60, 68]. One (3.8%) study each used MI+CBT [63]. In these studies, counseling focused on the importance of alcohol abstinence in pregnancy [54]; harm reduction with drink-size assessment [34], or health communication for healthy lifestyle [59]; brief advice to reduce alcohol intake [79]; non-stigmatizing counseling advising a reduction in alcohol consumption for women not able to abstain completely [76]; or a brief discussion with no specific recommendation on alcohol use [73]. Counseling in most of the studies (*n* = 10 studies, 38.5%) was provided by health professionals: in 4 (15.4%) studies by clinicians/ psychiatrics [55, 56, 60, 79], three studies by nurses (11.5%) [57, 65, 68], two studies by midwives (11.5%) [76, 78], one study (3.8%) by a nutritionist [63]; and in 10 studies by trained field researchers [58, 59, 61, 62, 66, 70–74]. In terms of the format of the intervention, three (11.5%) studies had self-administered computer-based counselling [64, 67, 77], and 3 (11.5%) studies had both intervention personnel and computer-based counseling [54, 69, 75]. Most studies (*n* = 14) included one single session varying in length from 5 to 60 minutes [54, 55, 59, 61–66, 69, 73, 74, 77, 79], (Additional File 3).

Of the 25 studies that had a control group, two studies provided controls with no screening or other treatment component at baseline, who were only screened at follow-up to record their change in alcohol use [74, 76]. The remaining 23 studies provided their control group with alcohol use screening at both baseline and at follow-up [54–69, 71–73, 75, 77–79]. Among these 23 studies, three provided only screening in their control groups with no other treatment component [54, 61, 65] while the remaining 20 (76.9%) studies had other treatment components in combination with the screening [55–60, 62–64, 66–69, 71–73, 75, 77–79]. In these studies, the control group received advice or counseling to abstain from or reduce alcohol use or to minimize the impact of drinking during pregnancy on the fetus by: healthcare staff in seven (26.9%) studies [56, 58, 63, 68, 75, 77, 78], or in the form of educational material in the form of brochure/videotape/manual in eight (30.8%) studies [55, 57, 58, 62, 66, 67, 71, 79], or received information regarding local places to assist them with alcohol management in two (11.5%) studies [57, 69]. Two studies mentioned providing usual care to the controls, but no detailed information was provided about the components [59, 73]. In fact, two (7.7%) studies received more extensive treatment than control groups in other studies [60, 72], at a level of intensity comparable to that of the intervention condition. In one of these studies, for example, the control group received at least 3 sessions of MET from clinicians that were 60 minutes or more in duration (same as the intervention), with the only difference being the intentional removal of some MET principles (e.g., avoiding confrontation, asking open-ended questions, reflective listening) [60]. In the other study, both the intervention and control groups were provided with the same CBT treatment (4 sessions, 7 minutes each), but the control group did not receive two post-intervention monitoring calls [72] (Additional File 3).

### Changes in antenatal alcohol use

Of 23 studies reporting change in alcohol use pre-post intervention (Additional Files 3 and 4), studies reported outcomes including: rates/odds of alcohol abstinence; odds/risks of alcohol use in pregnancy; mean drinks per week; number of drinking days; and mean differences in AUDIT scores. The data for baseline alcohol use of women across studies is heterogenous, reported using various methods: positive alcohol use screens (e.g., AUDIT, TWEAK T-ACE or MINI scores); alcohol use disorder diagnoses; past-month alcohol consumption; risk/binge/heavy drinking; drinks per week; alcohol dependence/use/abuse; and current alcohol use. Nine (36%) studies included populations of women with alcohol/substance use disorder or alcohol dependence, and one study (4%) focused exclusively on pregnant adolescents using alcohol. Six (24%) studies demonstrated significant reductions in alcohol use [55, 62, 63, 67, 71, 75]. A total of 17 of the 25 studies (68%) found no significant changes in alcohol use between BI and control groups [56–58, 60, 61, 64–66, 68, 69, 73–75, 77–79]. Notably, alcohol use decreased in both the control and BI groups in three studies [59, 72, 76]. One study involving adolescent pregnant women reported a substantial reduction in pre-post alcohol use in BI (22.3 to 13.1%) and controls (2.4 to 1.7%), without providing between group differences [59]. Another study without a control group found that pregnant women with heavy drinking showed a significant drop in mean drinks/week in the second trimester (8.6, *P* < 0.001), and third trimester (8.1, P < 0.001) after receiving BI compared to baseline (16.0) [70]. Finally, a study in Brazil found that both groups receiving BI with 2 weekly monitoring follow-up components (2 monitoring calls by the researcher in the first- and second-week post-intervention) versus those receiving BI without the monitoring component show higher reduction in mean-AUDIT, and mean T-ACE scores. No comparison for the change provided for between groups difference [72]. However, the percentages of abstinent pregnant women observed post-intervention were (92.3%) in the BI alone group compared to (100%) in the BI with monitoring component group. Regardless of the monitoring component, the study highlighted the importance of early intervention (from the first antenatal visit) in pregnancy to achieve significant reduction in prenatal alcohol use. Lastly, in several studies, effect measures for reduced alcohol use still favoured the intervention utilized, though these findings were not statistically significant [61, 64, 68, 69].

### Meta-analysis of alcohol abstinence post-intervention (BI v/s control)

**Fig. 2:**
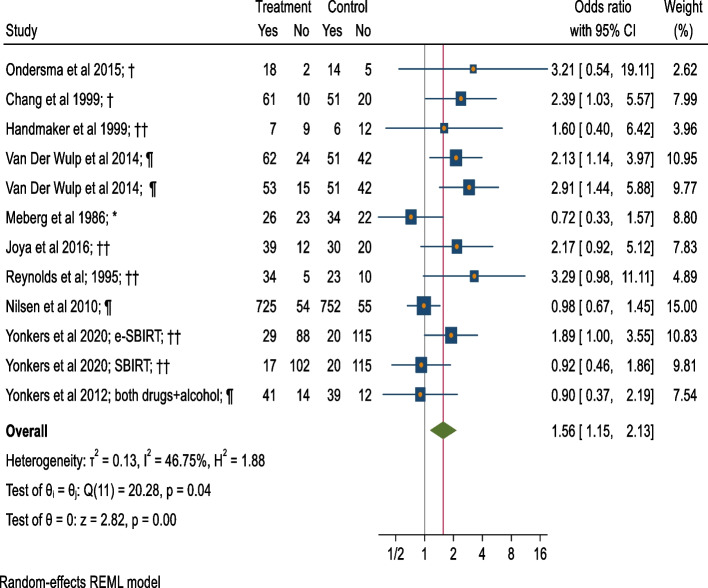
Forest plot of alcohol abstinence post intervention (BI vs Control). *: Screening + Motivational Interview/ Cognitive Behavioural Therapy/ Comprehensive counseling versus No treatment/ Control condition not explained. †: Screening + Motivational Interview/ Cognitive Behavioural Therapy/ Comprehensive counseling versus Screening. ††: Screening + Motivational Interview/ Cognitive Behavioural Therapy/ Comprehensive counseling versus Screening + Information on AU during pregnancy provision (verbal or oral). ¶: Screening + Extended-Motivational Interview/ Cognitive Behavioural Therapy/ Comprehensive counseling versus Screening + Information on AU during pregnancy provision (verbal or written). Extended interventions: Session/s lasting for more than 60 mins. in total or have more than 5 sessions

Meta-analyses of 12 BI arms versus control groups [57, 58, 61, 62, 64, 69, 75–78] for a combined total of 2620 pregnant women indicate that the BI group has 56% higher odds of being abstinent during pregnancy at any time-point (OR = 1.56, 95%CI = 1.15–2.13, moderate heterogeneity = 46.75%, *p* = 0.36) (Fig. 2; Table 1).

**Table 1:** Effect-sizes and other important statistics for alcohol use and neonatal outcome measures: Brief interventions versus Controls

Outcome Measures	Effect size measures	Randomized + Quasi experimental			Only Randomized			Split control groups with multiple arms		
		ES (95% CI)	I^**2**^; Tau^2^	***P***	ES (95% CI)	I^2^; Tau^2^	***P***	ES (95% CI)	I^2^; Tau^2^	*P*
Alcohol abstinence	OR	1.56* (1.15, 2.13)	46.75%; 0.13	0.04	1.86* (1.39, 2.49)	16.76%; 0.04	0.36	1.54* (1.12, 2.11)	40.92%; 0.12	0.08
	RR				1.23* (1.12, 1.35)					
Mean AUDIT scores	Hedge’s d	0.10 (−0.06, 0.26)	0.00%; 0.00	0.17	0.07 (− 0.09; 0.23)	0.00%; 0.00	0.87	NA	NA	NA
	UMD	0.13 (−0.07, 0.32)	0.00%; 0.00	0.09	0.09 (−0.11, 0.28)	0.00%; 0.00	0.93	NA	NA	NA
Mean drinks/week	Cohen’s d	−0.21 (−0.78, 0.36)	67.24%; 0.11	0.08	NA	NA	NA	−0.24 (− 0.86, 0.38)	64.41%; 0.13	0.09
	UMD	−0.10 (− 0.38, 0.19)	98.93%; 0.04	0.00	NA	NA	NA	−0.10 (− 0.38, 0.19)	98.33%; 0.04	0.00
Mean birth weight	Cohen’s d	0.16 (−0.36, 0.68)	81.40%; 0.17	0.00	0.131 (−0.74, 1.01)	87.43%; 0.35	0.00	NA	NA	NA
	UMD	76.74 (− 185.28, 338.76)	82.87%; 42,968.79	0.00	30.394 (− 332.09, 392.89)	88.92%; 60,964.71	0.00	NA	NA	NA
Low birthweight	OR	1.02 (0.44,2.40)	59.03%; 0.33	0.09	NA	NA	NA	0.93 (0.39, 2.20)	47.67%; 0.28	0.15
Preterm birth	OR	0.67* (0.46, 0.98)	0.00%; 0.00	0.58	NA	NA	NA	0.66 (0.43, 1.02)	0.00%; 0.00	0.60

*ES* Effect size, *NA* Not applicable, *OR* Odds ratio, *P*-value for test of homogeneity of study-specific effect sizes (> 0.05), *RR* Relative risk, *: Statistically significant effect size, I^2^ statistic (0 to 40%: might not be important, 30 to 60%: moderate heterogeneity, 50 to 90%: substantial heterogeneity, 75 to 100%: considerable heterogeneity), *UMD* Unstandardized Mean Difference

### Meta-analysis of mean AUDIT post-intervention (BI v/s control)

**Fig. 3:**
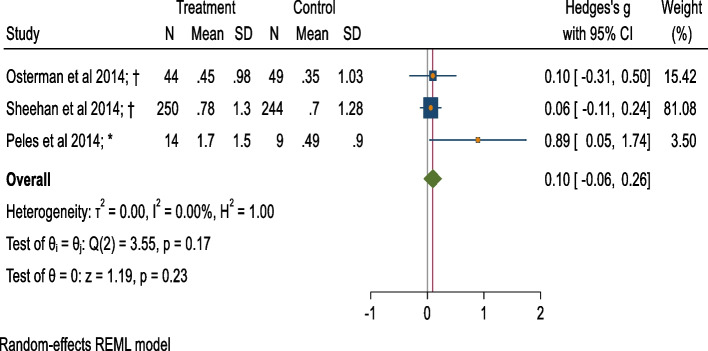
Forest plot of mean AUDIT scores post-intervention (BI v/s Control). *: Screening + Motivational Interview/ Cognitive Behavioural Therapy/ Comprehensive counseling versus No treatment/ Control condition not explained. † = Screening + Motivational Interview/ Cognitive Behavioural Therapy/ Comprehensive counseling versus screening

Three studies reported mean AUDIT scores [65, 73, 74] during pregnancy. The pooled estimates of mean AUDIT scores for a total of 610 pregnant women show a small and statistically insignificant difference between the BI group versus the control group (hedge’s g = 0.10, 95%CI = − 0.06 to 0.26, heterogeneity that might not be important = 0.0%, *p* = 0.17) (Fig. 3; Table 1).

### Meta-analysis of mean drinks/week

**Fig. 4:**
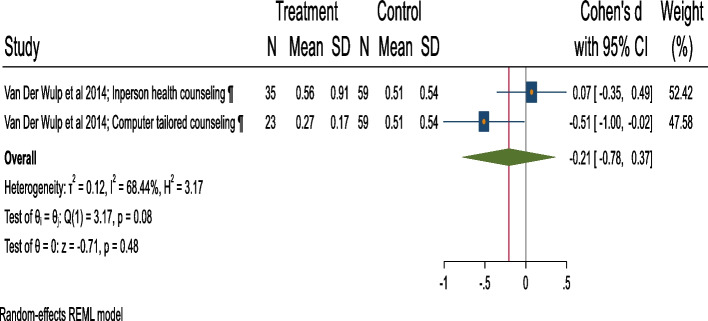
Forest plot of mean drinks/ week post-intervention (BI v/s control). ¶: Screening + Extended-Motivational Interview/ Cognitive Behavioural Therapy/ Comprehensive counseling versus Screening + Information on AU during pregnancy provision (verbal or written). Extended interventions: Session/s lasting for more than 60 mins. in total or have more than 5 sessions

The pooled estimates of 166 participants [75] (two intervention arms, one study) observed small and statistically insignificant difference in the mean drinks/week between BI versus control group (Cohen’s d = − 0.21, 95%CI = − 0.78 to 0.36, substantial heterogeneity =67.24%; *p* = 0.08) (Fig. 4; Table 1).

### Split-group analysis

No statistically significant differences in the effect sizes were observed when the control groups for multiple arms were split to obtain the effect estimates for alcohol abstinence and mean drinks per week when compared to the estimates obtained without splitting the control groups (Table 1).

### Subgroup and meta-regression analysis

There were fewer than the required number of studies for each alcohol use or neonatal outcome, therefore, it was not possible to conduct sub-group and meta-regression analyses.

### Sensitivity analyses: alcohol use outcomes

No statistically significant difference observed between the effect size estimate was obtained after excluding non-randomized control trials for prenatal alcohol abstinence when compared to their effect sizes including non-randomized trials in the meta-analysis (Table 1). However, the point estimate for odds ratio increased (although not statistically significant) and heterogeneity decreased (modest to might not be important) after excluding non-randomized trials from the meta-analysis for alcohol abstinence: 1.86 (95% CI = 1.93–2.49; heterogeneity might not be important = 16.76%; p = 0.08), (Table 1).

### Publication bias and small study effect

**Fig. 5:**
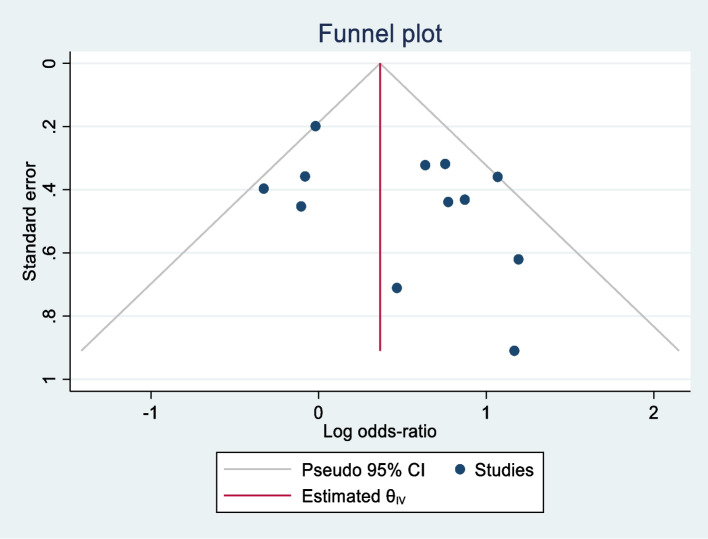
Funnel plot for publication bias for percentage of prenatal alcohol abstinence comparing BI v/s control

The funnel plot for the percentage of alcohol abstinence (Fig. 5) shows asymmetry indicating publication bias. However, the small study effect obtained from Peters test was not significant (*p* = 0.255), suggesting that smaller studies with larger effect size did not contribute significantly to the publication bias.

### Neonatal outcomes (BI versus control)

In total, seven studies reported neonatal outcomes [58, 65, 66, 75, 78]. The neonatal outcome measures reported in these studies were: preterm delivery [54]; NICU admission [54]; healthy pregnancy (live birth of ≥2500 g with no admission to NICU) [64]; mean difference in birth weight [66, 74]; mean difference in head circumference [66, 68]; body length; APGAR scores (1- or 5-minute) [55, 59, 74]; percentage of neonates born preterm [57], and LBW [54, 57]. A total of 3 studies showed significant difference in the neonatal outcomes between intervention and control groups, two in favour of the intervention and one in favour of the control [54, 66, 68].

It was not possible to conduct meta analysis due to insufficient number of studies or statistical information for the following outcomes: NICU admission; healthy pregnancy; head circumference; body length; APGAR scores; and percentage of neonates born preterm. Three studies in total reported on APGAR-1 and APGAR-5 scores [59, 61, 74]. Two studies found that in BI versus control groups, the mean APGAR-1 scores were: 8.7 (0.8) versus 8.1 (2) *P* = 0.1 [74]; and 8.1 versus 7.8 [61], respectively. Two studies found that in BI versus control groups, the mean APGAR-5 scores were: 8.9 versus 8.7 [61]; and 9.5 (1.1) versus 9.5 (1) *P* = 1.0 [74], respectively. In the third study, Sarvela and colleagues reported that APGAR scores in the BI versus control group were 8.7 (0.83) and 8.36 (1.42), respectively, however did not provide any *p* values [59].

### Meta-analysis of mean birth weight

**Fig. 6:**
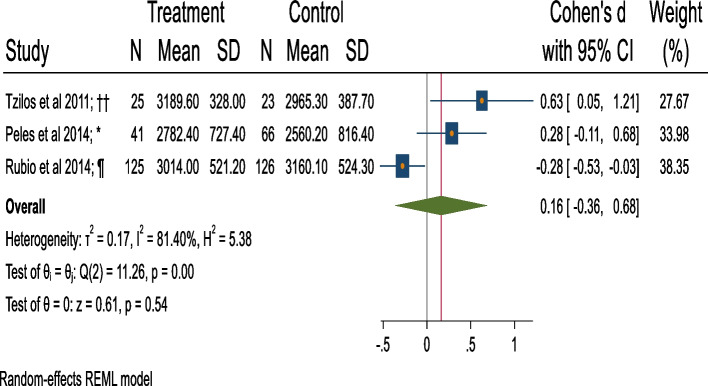
Forest plot of mean birth weight (BI v/s control). *: Screening + Motivational Interview/ Cognitive Behavioural Therapy/ Comprehensive counseling versus No treatment/ Control condition not explained. ††: Screening + Motivational Interview/ Cognitive Behavioural Therapy/ Comprehensive counseling versus Screening + Information on AU during pregnancy provision (verbal or oral). ¶: Screening + Extended-Motivational Interview/ Cognitive Behavioural Therapy/ Comprehensive counseling versus Screening + Information on AU during pregnancy provision (verbal or written). Extended interventions: Session/s lasting for more than 60 mins. in total or have more than 5 sessions

The pooled estimate of difference in the standardized mean difference in birth weight (grams) (Cohen’s d) [66, 68, 74] is small and statistically insignificant when comparing BI (*n* = 406) and control group (Cohen’s d = 0.16, 95%CI = − 0.36 to 0.68, with considerable – substantial heterogeneity = 81.40%; *p* = 0.00), and unstandardized mean difference (Fig. 6; Table 1).

### Meta-analysis of low birthweight

**Fig. 7:**
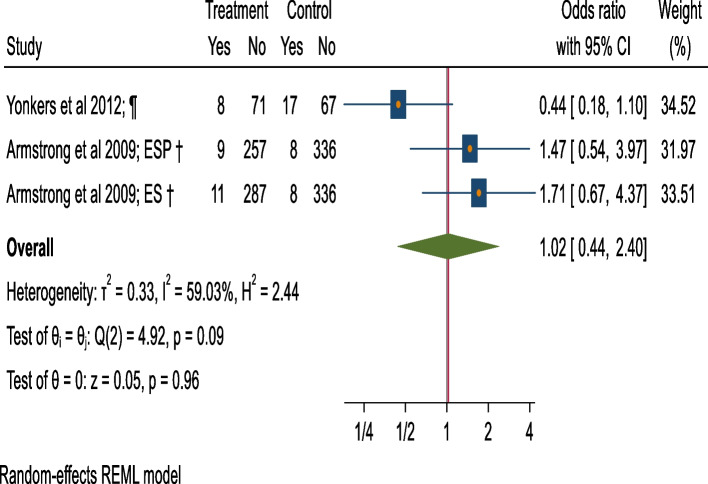
Forest plot of odds for low birth weight (BI v/s control). †: Screening + Motivational Interview/ Cognitive Behavioural Therapy/ Comprehensive counseling versus screening. ¶: Screening + Extended-Motivational Interview/ Cognitive Behavioural Therapy/ Comprehensive counseling versus Screening + Information on AU during pregnancy provision (verbal or written). Extended interventions: Session/s lasting for more than 60 mins. in total or have more than 5 sessions. ES: Early Start (Intervention group). ESP: Early Start Plus (Intervention group)

The pooled estimate of 2 studies [54, 57] for odds of LBW in the offspring of 1415 mothers studied does not show a significant difference between the BI (cases = 28) versus control group (cases = 33) (OR = 1.02, 95%CI = 0.44 to 2.40, moderate heterogeneity = 59.03%; *p* = 0.09), (Fig. 7; Table 1).

### Meta-analysis of preterm birth

**Fig. 8:**
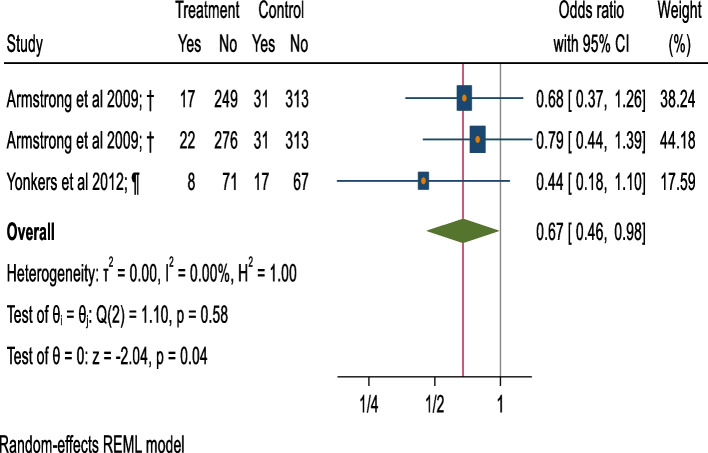
Forest plot of preterm birth (BI v/s control). †: Screening + Motivational Interview/ Cognitive Behavioural Therapy/ Comprehensive counseling versus screening. ¶: Screening + Extended-Motivational Interview/ Cognitive Behavioural Therapy/ Comprehensive counseling versus Screening + Information on AU during pregnancy provision (verbal or written). Extended interventions: Session/s lasting for more than 60 mins. in total or have more than 5 sessions

The meta-analysis for 3 intervention arms (2 studies) versus control groups, in 740 participants [54, 57] observe 33% lower odds of preterm birth among pregnant women in the intervention groups (cases = 47) compared to the control groups (cases = 79) (OR = 0.67, 95%CI = 0.46 to 0.98, small heterogeneity that might not be important = 0.00%; *p* = 0.58), (Fig. 8; Table 1).

### Split-group analysis: neonatal outcomes

The splitting of control groups in the multiple arms showed no statistically significant difference for low birth weight and preterm birth in BIs versus control groups when compared to the effect size estimates obtained when the control groups were not split (Table 1).

### Sensitivity analyses: neonatal outcomes

No statistically significant difference was observed between the effect size estimates obtained after excluding non-randomized control trials for mean birth weight when compared to their effect sizes when non-randomized trials were included in the meta-analysis (Table 1).

## Discussion

The current review and meta-analysis found that BIs were overall effective in increasing abstinence from alcohol during pregnancy. Results show that the odds of abstinence (56%) were significantly higher in pregnant women who received BIs compared to controls. However, despite small effects in the expected direction, no statistically significant difference was observed for studies examining changes in frequency of drinking (i.e., mean drinks per drinking day/week) and AUDIT scores. Control groups across all studies received at least a screening component, with a high degree of variability in additional education components, if any. Research indicates that even a single question about alcohol use has the potential to modify alcohol consumption in pregnancy [80]; therefore, it is possible that women in control groups reduced their intake due to receiving alcohol use screening, which may blur the effect of the intervention.

Abstinence from alcohol during pregnancy is the only way to completely avoid FASD in the child; however, complete abstention may not be possible for women with alcohol use disorders (AUDs), for example. In this review, nine of the studies focused on populations of pregnant women wherein a large portion screened positive for alcohol dependence or AUD, and the BIs utilized in these studies included goal-setting with both elimination and reduction components. All BIs for pregnant women, however, include public health messaging that abstaining completely is the only way to prevent FASD. More research is needed on BIs for pregnant women with AUDs or who have concurrent substance use disorders. Furthermore, in women with high pre-pregnancy drinking levels, prenatal care providers can impart additional family planning counselling in order to prevent high levels of PAE that may occur in early pregnancy.

For neonatal outcomes, it was found that pregnant women who received a BI had significantly lower odds (33% lower) of preterm birth when compared to the control groups, but no statistically significant differences were observed for APGAR score, mean birthweight, or LBW outcomes. While this study examines several adverse neonatal outcomes related to PAE, it is important to note that there are various chronic adverse effects of PAE that cannot be measured until early childhood or later in life, including changes in the brain structure and volume [81], immune system changes [82], and susceptibility to mental health disorders [83, 84], and FASD. Future research may examine the effects of BIs longitudinally, linking to outcomes in childhood, including the incidence of FASD and its common comorbidities (e.g., language disorders). Though there are many moderating factors for the adverse effects of PAE, such as nutrition and other substance use during pregnancy, it is worth nothing that preventing PAE significantly reduces the risk of many adverse health and social outcomes that are typically associated with FASD. Furthermore, preventing even one case of FASD incurs only 3% of the costs required to provide support services to an individual with FASD over their lifetime [85]; therefore, alcohol use screening and access to BIs in all formats should be prioritized in prenatal care services.

No studies were found on cost-effectiveness of BIs for pregnant women and, therefore, the review did not analyze these outcomes. It is worth noting, however, that BIs can be as short as five minutes in length and computer-based/digital formats may decrease resources required for service delivery [28]. Ultimately, access to BIs begins with screening for alcohol use, which is underutilized in prenatal care settings globally [86]. However, even a single question about alcohol use during pregnancy has immense potential to change a woman’s alcohol use behaviours [66, 80]. Women are generally accepting of alcohol use screening [87], and so it is important for care providers to use prenatal care visits as an opportunity to screen women and offer non-judgmental support in this efficient and low-cost manner. Furthermore, women can be referred to more intensive, effective programs that reduce maternal substance use, such as the Parent-Child Assistance Program (PCAP), which has proven to be cost-effective [88].

These findings are in line with previous reviews on BIs for alcohol use in pregnant women. For example, Erng et al.’s systematic review of interventions seeking to prevent alcohol-related harm during pregnancy [89] also found some support, although inconsistent, for alcohol-focused BIs in pregnancy. The meta-analysis by Gomez and colleagues [28] found stronger support for psychosocial interventions for alcohol use during pregnancy than we report here, but included a broader range of intervention types, included qualitative analysis of treatment components and focused on BIs for both pregnant and postpartum women. Three key aspects of this literature merit highlighting. First, studies in this area are highly variable in inclusion criteria, intervention characteristics (including dose and duration), outcome measures, follow-up duration, and in the extent to which key details are reported. These factors certainly contribute to the inconsistency of results seen in the reviewed studies. In addition, the heterogeneity in study populations contributes to the overall assessment of low certainty in the evidence using the GRADE approach. Second, this area is marked by a lack of rigorous research seeking to identify subgroups that might respond best to BIs, or seeking to identify the key behavior change techniques, duration, or frequency needed to obtain stable effects on alcohol in pregnancy. Early work of this type has suggested that two sessions may be more effective than a single session [90], which if true would mirror the tobacco brief intervention literature [91]. Third, the relatively small effects seen with BIs means that larger samples will be crucial for clearly identifying any positive BI effects.

### Strengths and limitations

This review has several notable strengths, including its inclusion of a wide range of studies in multiple languages to reduce bias, its inclusion and analysis of various outcome measures, its detailed meta-analyses, as well as its extension of previous literature by including neonatal outcomes from BIs. This study also has several limitations. Firstly, across all studies included, there was a high within-group variation among both BI groups and control groups, in terms of their components, educational content, number of sessions, and duration of intervention. Studies utilized a variety of tools (*n* = 4) to screen alcohol use among pregnant women, with varying sensitivity, specificity and overall clinical utility, and even different approaches to scoring [86]. Each of these tools can be administered in-person and online and may be subject to certain biases, such as social desirability bias [86]. Moreover, the baseline characteristics of pregnant women were also variable in terms of their biological age, gestational age, and levels of alcohol consumption. Due to a limited number of studies, it was not possible to conduct sub-group analysis to explore factors influencing the heterogeneity. For this reason, it is not possible to draw conclusions about which sub-populations of pregnant women may benefit most from specific formats or techniques used in BIs for alcohol use.

## Conclusions

Based on the findings from this study, we can conclude that BIs are moderately effective in increasing abstinence during pregnancy and may also be modestly effective at preventing preterm births among infants at high risk for PAE. More studies on the effectiveness of BIs in alcohol use in pregnant women are needed from low- and middle-income countries, as well as among younger mothers, and some subpopulations who are at high risk for alcohol use during pregnancy. There is also a clear need for rigorous research seeking to optimize BI efficacy, in part by exploring subgroups that are most likely to benefit from these interventions.

## Supplementary Information

**Additional file 1.** Systematic literature search strategy.

**Additional file 2.** PRISMA Checklist.

**Additional file 3.** Characteristics of studies included in the systematic review.

**Additional file 4.** Screening tools and outcome measures.

**Additional file 5.** Quality assessment of studies included in the systematic review.

**Additional file 6.** Assessment in the certainty of evidence for each outcome included in the meta-analysis using the GRADE Approach.

## Data Availability

The datasets used and/or analysed during the current study available from the corresponding author on reasonable request.

## Abbreviations

APGAR: Appearance, Pulse, Grimace, Activity, and Respiration
AUD: Alcohol use disorder
AUDIT: Alcohol Use Disorders Identification Test
BI: Brief intervention
CBT: Cognitive behavioural therapy
CEBM: Centre for Evidence Based Medicine
C-RCT: Cluster randomized control trial
FASD: Fetal Alcohol Spectrum Disorder
IUGR: Intrauterine growth retardation
LBW: Low birth weight
MET: Motivational Enhancement Therapy
MI: Motivational interviewing
NICU: Neonatal intensive care unit
OR: Odds ratio
PAE: Prenatal alcohol exposure
RCT: Randomized control trials
SBI: Screening and Brief Intervention
SBIRT: Screening, Brief Intervention, and Referral to Treatment
TLFB: Timeline follow back
WHO: World Health Organization
