# Supplementary material for: Effectiveness of brief alcohol interventions for pregnant women: a systematic literature review and meta-analysis
Source: BMC Pregnancy Childbirth. 2023 Jan 24;23:61. doi: 10.1186/s12884-023-05344-8 (PMC9872314; doi:10.1186/s12884-023-05344-8)
Supplement: Supplementary file 1 — Additional file 1. Systematic literature search strategy. [file 12884_2023_5344_MOESM1_ESM.docx]

**Additional File 1. Systematic literature search strategy**

1. **Medline Ovid (All)**

| [# ▲](http://ovidsp.dc2.ovid.com.myaccess.library.utoronto.ca/ovid-b/ovidweb.cgi?&S=KOOPFPLHAKEBFDPBJPAKHFBHFCDAAA00&Sort+Sets=descending) | **Searches** | **Results** |
| --- | --- | --- |
| 1 | exp Female/ or reproductive female*.mp. | 8841734 |
| 2 | exp Pregnancy/ or exp Middle Aged/ or exp Adolescent/ or reproductive age.mp. or exp Adult/ or exp Young Adult/ | 8504075 |
| 3 | exp Female/ or exp Adolescent/ or exp Pregnancy/ or exp Middle Aged/ or child-bearing age.mp. or exp Adult/ or exp Young Adult/ | 10921280 |
| 4 | pregnant.mp. or exp A) PW/ | 189645 |
| 5 | exp Pregnancy/ or Gestation*.mp. | 988574 |
| 6 | exp Pregnancy Trimester, Second/ or trimester.mp. or exp Pregnancy Trimester, First/ or exp Pregnancy Trimester, Third/ | 73892 |
| 7 | exp drinking behavior/ or exp alcohol drinking/ | 76580 |
| 8 | (alcohol adj3 (drink$ or intoxicat$ or use$ or abus$ or misus$ or risk$ or consum$ or withdraw$ or detox$ or treat$ or therap$ or excess $ or reduc$ or cessation or intervention$)).tw. | 130335 |
| 9 | exp alcohol-related disorders/ or exp alcoholism/ | 113755 |
| 10 | (drink$ adj3 (excess or heavy or heavily or harm or harmful or hazard$ or binge or harmful or problem$)).tw. | 20645 |
| 11 | exp Counseling/ | 44605 |
| 12 | exp Psychotherapy/ | 198143 |
| 13 | exp Cognitive Behavioral Therapy/ | 29396 |
| 14 | exp Interview, Psychological/ | 15108 |
| 15 | exp Motivational Interviewing/ | 1945 |
| 16 | ((brief or minimal or early or motivat$) adj3 (intervention$ or therap$ or interview$ or advice)).tw. | 76600 |
| 17 | (counselling or counseling or advice).tw. | 142251 |
| 18 | exp "randomized controlled trial (topic)"/ | 0 |
| 19 | exp time series analysis/ | 0 |
| 20 | exp clinical trial/ or exp controlled clinical trial/ or exp randomized controlled trial/ or exp pragmatic trial/ | 877028 |
| 21 | 1 or 2 or 3 or 4 or 5 or 6 | 11000268 |
| 22 | 7 or 8 or 9 or 10 | 235534 |
| 23 | 11 or 12 or 13 or 14 or 15 or 16 or 17 or 18 or 19 or 20 | 1266139 |
| 24 | limit 21 to (human and yr="1987 -Current") | 7867927 |
| 25 | limit 22 to (human and yr="1987 -Current") | 153599 |
| 26 | limit 23 to (human and yr="1987 -Current") | 1041582 |
| 27 | 21 and 22 and 23 | 17140 |
| 28 | limit 27 to yr="1987 -Current" | 15063 |
| 29 | limit 28 to (humans and yr="1987 -Current") | 14992 |

|  |
| --- |

1. **Embase**

| \| [# ▲](http://ovidsp.dc2.ovid.com.myaccess.library.utoronto.ca/ovid-b/ovidweb.cgi?&S=KOOPFPLHAKEBFDPBJPAKHFBHFCDAAA00&Sort+Sets=descending) \| **Searches** \| **Results** \| \| --- \| --- \| --- \| \| 1 \| exp Female/ or reproductive female*.mp. \| 10108696 \| \| 2 \| exp Pregnancy/ or exp Middle Aged/ or exp Adolescent/ or reproductive age.mp. or exp Adult/ or exp Young Adult/ \| 10414922 \| \| 3 \| exp Female/ or exp Adolescent/ or exp Pregnancy/ or exp Middle Aged/ or child-bearing age.mp. or exp Adult/ or exp Young Adult/ \| 13206401 \| \| 4 \| pregnant.mp. or exp A) PW \| 282466 \| \| 5 \| exp Pregnancy/ or Gestation*.mp. \| 1001316 \| \| 6 \| exp Pregnancy Trimester, Second/ or trimester.mp. or exp Pregnancy Trimester, First/ or exp Pregnancy Trimester, Third/ \| 112808 \| \| 7 \| exp drinking behavior/ or exp alcohol drinking/ \| 51778 \| \| 8 \| (alcohol adj3 (drink$ or intoxicat$ or use$ or abus$ or misus$ or risk$ or consum$ or withdraw$ or detox$ or treat$ or therap$ or excess$ or reduc$ or cessation or intervention$)).tw. \| 186933 \| \| 9 \| exp alcohol-related disorders/ or exp alcoholism/ \| 135733 \| \| 10 \| (drink$ adj3 (excess or heavy or heavily or harm or harmful or hazard$ or binge or harmful or problem$)).tw. \| 28662 \| \| 11 \| exp Counseling/ \| 176553 \| \| 12 \| exp Psychotherapy/ \| 280268 \| \| 13 \| exp Cognitive Behavioral Therapy/ \| 14650 \| \| 14 \| exp Interview, Psychological/ \| 871 \| \| 15 \| exp Motivational Interviewing/ \| 5173 \| \| 16 \| ((brief or minimal or early or motivat$) adj3 (intervention$ or therap$ or interview$ or advice)).tw. \| 116446 \| \| 17 \| (counselling or counseling or advice).tw. \| 212094 \| \| 18 \| exp "randomized controlled trial (topic)"/ \| 192325 \| \| 19 \| exp time series analysis/ \| 27960 \| \| 20 \| exp clinical trial/ or exp controlled clinical trial/ or exp randomized controlled trial/ or exp pragmatic trial/ \| 1575966 \| \| 21 \| 1 or 2 or 3 or 4 or 5 or 6 \| 13296290 \| \| 22 \| 7 or 8 or 9 or 10 \| 296235 \| \| 23 \| 11 or 12 or 13 or 14 or 15 or 16 or 17 or 18 or 19 or 20 \| 2354836 \| \| 24 \| limit 21 to (human and yr="1987 -Current") \| 10452112 \| \| 25 \| limit 22 to (human and yr="1987 -Current") \| 215090 \| \| 26 \| limit 23 to (human and yr="1987 -Current") \| 2112651 \| \| 27 \| 21 and 22 and 23 \| 21522 \| |
| --- | --- | --- | --- | --- | --- | --- | --- | --- | --- | --- | --- | --- | --- | --- | --- | --- | --- | --- | --- | --- | --- | --- | --- | --- | --- | --- | --- | --- | --- | --- | --- | --- | --- | --- | --- | --- | --- | --- | --- | --- | --- | --- | --- | --- | --- | --- | --- | --- | --- | --- | --- | --- | --- | --- | --- | --- | --- | --- | --- | --- | --- | --- | --- | --- | --- | --- | --- | --- | --- | --- | --- | --- | --- | --- | --- | --- | --- | --- | --- | --- | --- | --- | --- | --- |

1. **PsycINFO:**

| \|  \| [# ▼](http://ovidsp.dc2.ovid.com.myaccess.library.utoronto.ca/ovid-b/ovidweb.cgi?&S=KOOPFPLHAKEBFDPBJPAKHFBHFCDAAA00&Sort+Sets=ascending) \| **Searches** \| **Results** \| \| --- \| --- \| --- \| --- \| \|  \| 23 \| limit 22 to (human and yr="1987 -Current") \| 967 \| \|  \| 22 \| 19 and 20 and 21 \| 1078 \| \|  \| 21 \| 11 or 12 or 13 or 14 or 15 or 16 or 17 or 18 \| 380208 \| \|  \| 20 \| 4 or 5 or 6 or 7 or 8 or 9 or 10 \| 117889 \| \|  \| 19 \| 1 or 2 or 3 \| 187062 \| \|  \| 18 \| exp Treatment Effectiveness Evaluation/ or exp Cognitive Behavior Therapy/ or exp Clinical Trials/ or exp Health Promotion/ or exp Intervention/ or exp Prevention/ or randomized control trials.mp. \| 224147 \| \|  \| 17 \| exp Alcohol Treatment/ or exp Intervention/ or brief alcohol intervention$.mp. \| 118582 \| \|  \| 16 \| (counselling or counseling or advice).mp. [mp=title, abstract, heading word, table of contents, key concepts, original title, tests & measures, mesh] \| 125617 \| \|  \| 15 \| ((brief or minimal or early or motivat$) adj3 (intervention$ or therap$ or interview$)).mp. [mp=title, abstract, heading word, table of contents, key concepts, original title, tests & measures, mesh] \| 46190 \| \|  \| 14 \| cognitive behavior therapy/ or behavior therapy/ \| 33969 \| \|  \| 13 \| exp Motivational Interviewing/ \| 2531 \| \|  \| 12 \| exp Brief Psychotherapy/ \| 5645 \| \|  \| 11 \| counseling/ or rehabilitation counseling/ \| 24904 \| \|  \| 10 \| ("AU" or alcoholic$).mp. [mp=title, abstract, heading word, table of contents, key concepts, original title, tests & measures, mesh] \| 63733 \| \|  \| 9 \| (drink$ adj3 (excess or heavy or heavily or hazard$ or binge or harmful or problem$)).mp. [mp=title, abstract, heading word, table of contents, key concepts, original title, tests & measures, mesh] \| 17578 \| \|  \| 8 \| (alcohol adj3 (drink$ or intoxicat$ or use$ or abus$ or misus$ or risk$ or consum$ or withdraw$ or detox$ or treat$ or therap$ or excess $ or reduc$ or cessation or intervention)).mp. [mp=title, abstract, heading word, table of contents, key concepts, original title, tests & measures, mesh] \| 96333 \| \|  \| 7 \| exp Alcohol Drinking Patterns/ \| 26222 \| \|  \| 6 \| alcohol rehabilitation.mp. or exp Alcohol Treatment/ \| 9586 \| \|  \| 5 \| exp Alcohol Abuse/ \| 48977 \| \|  \| 4 \| exp Alcohol Intoxication/ \| 3238 \| \|  \| 3 \| exp Pregnancy/ or exp Perinatal Period/ or pregnancy trimester.mp. \| 44673 \| \|  \| 2 \| exp Pregnancy/ or exp Mothers/ or gestation*.mp. \| 85276 \| \|  \| 1 \| exp Human Women/ or exp Pregnancy/ or reproductive age.mp. \| 179290 \| |
| --- | --- | --- | --- | --- | --- | --- | --- | --- | --- | --- | --- | --- | --- | --- | --- | --- | --- | --- | --- | --- | --- | --- | --- | --- | --- | --- | --- | --- | --- | --- | --- | --- | --- | --- | --- | --- | --- | --- | --- | --- | --- | --- | --- | --- | --- | --- | --- | --- | --- | --- | --- | --- | --- | --- | --- | --- | --- | --- | --- | --- | --- | --- | --- | --- | --- | --- | --- | --- | --- | --- | --- | --- | --- | --- | --- | --- | --- | --- | --- | --- | --- | --- | --- | --- | --- | --- | --- | --- | --- | --- | --- | --- | --- | --- | --- | --- |

1. **CINAHL: 3933**

| **Search Terms** | **Search Options** |
| --- | --- |
| S25 | S22 AND S23 AND S24  **Limiters** - Published Date: 19870101-20201231; Sex: Female; Age Groups: Fetus, Conception to Birth, Infant, Newborn: birth-1 month, Infant: 1-23 months, Adolescent: 13-18 years, Adult: 19-44 years, Middle Aged: 45-64 years  **Search modes** - Boolean/Phrase |
| S24 | S13 OR S14 OR S15 OR S16 OR S17 OR S18 OR S19 OR S20 OR S21 |
| S23 | S9 OR S10 OR S11 OR S12 |
| S22 | S1 OR S2 OR S3 OR S4 OR S5 OR S6 OR S7 OR S8 |
| S21 | (MH "Counseling") OR (MH "Motivational Interviewing") |
| S20 | TI ((brief N3 intervention*) OR (brief N3 therap*) OR (brief N3 interview*) OR (minimal N3 intervention*) OR (minimal N3 therap*) OR (minimal N3 interview*) OR (early N3 intervention*) OR (early N3 therap*) OR (early N3 interview*) OR (motivat* N3 intervention*) OR (motivat* N3 therap*) OR (motivat* N3 interview*)) OR AB ((brief N3 intervention*) OR (brief N3 therap*) OR (brief N3 interview*) OR (minimal N3 intervention*) OR (minimal N3 therap*) OR (minimal N3 interview*) OR (early N3 interven [...](javascript:showHistoryTerm('ctl00_ctl00_FindField_FindField_historyControl_HistoryRepeater_ctl05_ellipsis',true)) |
| S19 | TI (counseling OR counselling OR advice) OR AB (counseling OR counselling OR advice) |
| S18 | TI ("brief alcohol intervention*") OR AB ("brief alcohol intervention*") |
| S17 | (MH "Behavior Therapy+") |
| S16 | (MM "Psychotherapy, Brief") |
| S15 | (MM "Intervention Trials") OR (MH "Early Intervention+") OR (MM "Internet-Based Intervention") OR (MM "Experimental Studies+") OR "brief interventions" |
| S14 | (MM "Time Series") OR (MM "Interrupted Time Series Analysis") OR (MM "Multiple Time Series") |
| S13 | (MH "Randomized Controlled Trials+") OR (MH "Clinical Trials+") OR (MM "Cochrane Library") |
| S12 | (MH "Alcohol Drinking+") OR (MH "Substance Withdrawal Syndrome+") OR (MH "Alcohol-Induced Disorders, Nervous System+") OR (MM "Alcohol Drinking in College") OR (MM "Alcohol Abuse (Saba CCC)") OR (MM "Alcohol Abuse Control (Saba CCC)") OR (MM "Alcohol Withdrawal Syndrome") OR (MH "Substance Use Rehabilitation Programs+") OR (MM "Substance Use Treatment: Alcohol Withdrawal (Iowa NIC)") OR (MM "Substance Withdrawal, Controlled") |
| S11 | (MH "Alcohol Abuse+") |
| S10 | (MH "Alcoholic Intoxication+") OR (MH "Alcohol Rehabilitation Programs+") OR (MM "Alcoholism") |
| S9 | (MM "Alcoholism") OR "alcoholism" OR (MH "Alcohol Abuse+") |
| S8 | (MH "Pregnancy Trimesters+") |
| S7 | (MM "Middle Age") |
| S6 | (MM "Young Adult") |
| S5 | "child-bearing" |
| S4 | (MH "Pregnancy+") |
| S3 | (MH "Gestational Age") OR (MH "Maternal Age") OR (MH "Maternal Age 35 and Over") OR (MH "Maternal Age 14 and Under") OR "reproductive age" |
| S2 | MH reproductive age |
| S1 | (MM "Female") OR "female" |

1. **Web of science: SCI-expanded, CPSI-S; ESCI**

| **Set** | **Results** | **Save History / Create Alert Open Saved History** |
| --- | --- | --- |
| # 6 | [702](http://apps.webofknowledge.com/summary.do?product=WOS&doc=1&qid=6&SID=7FRPIUhMl58gnKs6q6I&search_mode=CombineSearches&update_back2search_link_param=yes) | #5 AND #3 AND #2  *Indexes=SCI-EXPANDED, SSCI, ESCI Timespan=All years* |
| # 5 | [1,580,224](http://apps.webofknowledge.com/summary.do?product=WOS&doc=1&qid=5&SID=7FRPIUhMl58gnKs6q6I&search_mode=CombineSearches&update_back2search_link_param=yes) | #4 OR #1  *Indexes=SCI-EXPANDED, SSCI, ESCI Timespan=All years* |
| # 4 | [539,281](http://apps.webofknowledge.com/summary.do?product=WOS&doc=1&qid=4&SID=7FRPIUhMl58gnKs6q6I&search_mode=AdvancedSearch&update_back2search_link_param=yes) | ts=(pregnan*)  *Indexes=SCI-EXPANDED, SSCI, ESCI Timespan=All years* |
| # 3 | [26,573](http://apps.webofknowledge.com/summary.do?product=WOS&doc=1&qid=3&SID=7FRPIUhMl58gnKs6q6I&search_mode=AdvancedSearch&update_back2search_link_param=yes) | ts=(brief intervention)  *Indexes=SCI-EXPANDED, SSCI, ESCI Timespan=All years* |
| # 2 | [613,144](http://apps.webofknowledge.com/summary.do?product=WOS&doc=1&qid=2&SID=7FRPIUhMl58gnKs6q6I&search_mode=AdvancedSearch&update_back2search_link_param=yes) | ts=(alcohol*)  *Indexes=SCI-EXPANDED, SSCI, ESCI Timespan=All years* |
| # 1 | [1,089,584](http://apps.webofknowledge.com/summary.do?product=WOS&doc=1&qid=1&SID=7FRPIUhMl58gnKs6q6I&search_mode=AdvancedSearch&update_back2search_link_param=yes) | ts=(female*)  *Indexes=SCI-EXPANDED, SSCI, ESCI Timespan=All years* |

1. [ichgcp.clinical_trials.find](https://ichgcp.net/clinical-trials-registry/research/find)

(<https://ichgcp.net/clinical-trials-registry/research/find?term=Alcohol&recr=&rslt=&type=&cond=&intr=brief+interventions&outc=&spons=&lead=&id=&state1=&cntry1=&state2=&cntry2=&state3=&cntry3=&locn=&gndr=Female&rcv_s=&rcv_e=&lup_s=&lup_e=>): 405 results

1. google scholar: 17 600

alcohol women brief intervention drinking OR alcoholism OR binge OR addiction "brief intervention" Time: 1987-2020
