# Supplementary material for: Effectiveness of brief alcohol interventions for pregnant women: a systematic literature review and meta-analysis
Source: BMC Pregnancy Childbirth. 2023 Jan 24;23:61. doi: 10.1186/s12884-023-05344-8 (PMC9872314; doi:10.1186/s12884-023-05344-8)
Supplement: Supplementary file 3 — Additional file 3. Characteristics of studies included in the systematic review. [file 12884_2023_5344_MOESM3_ESM.docx]

**Additional File 3. Characteristics of studies included in the systematic review**

| **Reference, country, setting, design** | **Baseline:**  **a) Alcohol/SU;**  **b) Gestational age** | **a) Age (years); b) Ethnicity/ Race; c) Income; d) Education; e) Marital status; f) Employment; g) Total participants (N)** | **Content of intervention: a) Administered by; b) Number of sessions; c) Duration d) Time of FU questionnaire** | **Control group** | **Outcomes**  **(BI v/s Control)** | **Neonatal outcomes**  **(BI v/s Control)** |
| --- | --- | --- | --- | --- | --- | --- |
| 1. (1) Armstrong et al., 2009;   USA, Northern CA;  Obstetric clinic;  C-RCT | 1. PW tested positive for AU 2. Median (IQR)= 21 (17–27) wks. | 1. (ESP; ES; C) 15–21 yrs= 29, 42, 24; 22–25 yrs=25, 18, 24; 26–30 yrs= 18, 20, 27; 31–44 yrs = 29, 20, 24. 2. White: 29, 45, 65; Black: 16, 9, 4; Hispanic: 32, 24, 11; Asian: 9, 4, 3; Other: 14, 18, 17. 3. Annual income (<$25,000): 36, 44, 25. 4. Education (<high school): 47, 45, 31. 5. Married/partner: 59, 60, 75 6. n/a 7. N= 908 | Two arms:   1. 1) ES: In person 2. screening + counseling focused on abstention 3. 2) ESP: Computerized   screening + counseling focused on harm-reduction with computerized add-on Drink size assessment.  1) ES: a) licensed clinical social workers, or other trained substance- abuse treatment providers; b) 1; c) mins. n/a; d) Later in pregnancy (exact time not specified)  2) ESP: a) Computer; b) 1; c) 20-30 min; d) Later in pregnancy- no specific time mentioned | - Screening | n/a | - 1) OR Assisted Ventilation: - ESP v/s C; ES v/s C; ESP v/s ES: NS - 2) OR LBW (<2500 gm): **ESP v/s C; ES v/s C= 0.28 (0.10–0.80; 0.02); ESP v/s ES= NS - 3) OR Preterm delivery (<35 wks.): NS difference b/w any groups - 4) OR NICU admission: NS difference b/w any groups - 5) OR Rehospitalization: NS difference b/w any groups |
| (2) Chang et al., 1999  USA, Boston, MA;  Hospital-based prenatal clinic;  RCT | 1. PW who drinks   Life-time DSM-III-R alcohol diagnoses: I= 43%, C= 38%   1. M(SD)= =16(4.6) wks. | (T)   1. M(SD, Range)= 30.7(5.4, 18-43) yrs 2. n/a 3. n/a 4. n/a 5. n/a 6. n/a 7. N= 250 | Screening + CBT/ MI, - focused on abstinence.  MI-CBT content: reviewing participant's health + lifestyle changes made since pregnancy, goal setting, risk situation and alternatives to prevent alcohol related situations, summary of the key points in take home manual  a) RA; b) 1; c) 45 mins.; d) post-partum | - Screening | - RR for AUP (BI v/s control) = 0.80, P=0.33: NS; - APGAR-1 and 5: NS difference b/w groups | - Neonatal outcome: Birth weight (BI v/s control) = 3360 v/s 3406 grams. APGAR-1 = (8.1 v/s 7.8). APGAR-5= (8.9 v/s 8.7), |
| (3) Handmaker et al., 1999;   1. USA, Albuquerque, NM;   Obstetrics clinic (academic/university medical centre), outpatient psychology clinic or patient homes;  RCT | 1. PW who consumed ≥ 1 drink in past month. 2. Anytime during pregnancy | (T)  a) M(SD)= 24 (5.76)  b) (%): Hispanic=53, White= 38, Black= 9%  c) n/a  d) M (SD) yrs of education = 12.0 (2.71)  e) Unmarried (%): 62  f) Unemployed (%): 50  g) N= n/a | Screening+ MI encouraging them to change for better outcomes of quitting on their fetus  MI content:  a) Assess participants’ understanding on AUP on fetus; b) Feed-back by charting their level of drinking on various stages of their fetus development, and encouragement to quit at any point  a) RA; b) 1; c) 60 mins; d) 2 months post-enrolment | Screening + Structured interview using the (BDP) + Education: letters informing about potential risks of AUP and referring them to their health care provider, not in-person | Effect size θ - (standardized M difference b/w BI v/s Controls): within group difference is comparable b/w BI (θ=0.40; 0.46; 0.20) and control (θ=0.46, 0.77, 0.69), for changes in consumption, BAC, and abstinence, respectively.  ***** The effect ranges from small to medium. | n/a |
| 1. (4) O'Connor & Whaley et al., 2007;   USA, LA & OC, CA;  Community-based;  C-RCT | 1. PW with AU following pregnancy recognition.   M(SD)= TWEAK score: C= 1.84 (1.54), I= 1.77 (1.36)   1. M 17.98(7.87) wks. | (T)  a) M(SD)= 28.18 (5.97)  b) (%): White= 7.1, Black= 17.3, English speaking Hispanic= 26.3; Spanish speaking Hispanic= 44.3  c) Earn 15,000 or less (%)= 67  d) M(SD) years of education= 11.09 (3.42)  e) Married or has a partner (%)= 71.4  f) n/a  g) 255 | Screening + CBT; MI using workbook- focused on abstinence.  MI framework: a) feedback, b) cognitive behavioral procedures, c) goal setting, and contracting  Standardized workbook: feedback on current health behaviors, a review of the prevalence of problem drinking, a list of the adverse effects of alcohol, a worksheet on drinking cues, a drinking agreement in the form of a prescription, and drinking diary cards.  a) Nutritionist; b) 1; c) 10-15 mins.; d) 3^rd^ trimester | Screening + BA to avoid AUP | ******Odds Ratio for abstinent (OR=5.39; 95% CI = 1.59 - 18.25).  Odds of being abstinence 5.39 times higher if received a BI compared to control | n/a |
| 1. (5) Ondersma et al., 2015;   USA, Detroit, MIS ;  Prenatal clinic ;  RCT | a) PW risky drinkers. Binge drinking ≥ 1/ week)= 58.3%; Alcohol dependence/ abuse= 25%  b) M 12.2 wks. | - (T)   a) (%): 18-25 yrs= 54.2, 26-33 yrs= 33.3, 34-37 yrs= 12.5  b) African American (%)=81.3  c) n/a  d) n/a  e) Married (%)= 20.8  f) n/a  g) N= 48 | Screening + MI based on the participant’s goals and ability to quit; reduction, abstinence, or relapse prevention by brief testimonial videos + Tailored mailing at regular intervals   - MI framework: SDT sought to facilitate self-change and/or treatment-seeking, Decisional balance, feedback, and optional goal setting   Brief videos by physician on effects of AEP - 3 different versions of mothers’ testimonies: 3 branches on BI: those not willing to quit, intending to change, and who have quitted since knowing the pregnancy but can relapse  a) Computer; b) 1; c) 20 mins.; d) at-delivery | - Screening + moderately interactive intervention focused on infant nutrition, with no mention of AUP- 20 mins. | - OR: 90-day period prevalence abstinence= aOR: 3.4 (0.5-21.0): NS | - n/a |
| (6) Osterman et al., 2014;  USA, Cincinnati, OH;  Prenatal clinic (in a University medical centre);  RCT | a) PW who reported any AU in the previous year.  M (SD) AUDIT scores: I= 4.86 (4.99), C= 5.60 (4.87)  b) M= 24.3 wks | - (I, C) - a) M(SD): I= 25.27(4.67); C= 25.55 (4.98) - b) Black, White, Multi-racial (%): I=: 61.3, 27.4, 6.5; C= 55, 33.3, 5 - c) <15k, 15k-30k, 30.1k-45k, 45.1k-60k, 60.1k-75k (per annum) (%): I= 71, 16.1, 1.6, 1.6, 1.6.; C= 68.3, 20, 3.3, 0, 1.7 - d) < 12 years, high school graduate, 4 year college graduate, graduate degree (%): I= 24.2, 24.2, 42, 1.6, 1.6; C= 26.7, 25, 41.7, 3.3, 0 - e) Married, Single, Divorced (%): I= 16.1, 77.4, 3.2; C= 13.3, 83.3, 0 - f) n/a - g) N= 122 | Screening + MI, in-person- focused on reducing AU  MI framework SDT: need for relatedness, autonomy, competence); 4 Principles of MI-establishing empathy, developing discrepancy, rolling with resistance, supporting self-efficacy.  a) An adult psychiatric mental health clinical nurse specialist with specialty certification; b) 1; c) 30 mins.; d) 30 days post-intervention and 30 days post-partum | - Screening | BI v/s control comparable in M drinks/ week, M drinks/day, and M AUDIT scores at 30 days post-baseline and 30-days post-partum | n/a |
| (7) Sheehan et al., 2014;  Ireland, Dublin;  Hospital-based;  RCT | a) PW consuming alcohol  Mean AUDIT (SD): C= 0.9(1.5), I= 1.0(1.6)  b) M = 16.1 wks. | - (T) - a) M(SD, Range)=27.8 (5.7; 18-46) yrs - b) Ireland; EU; Asia/ Australasia, Africa, Europe but not EU, Americans (%): 76.4, 16.9, 2.6, 2.3, 1.2, 0.6 - c) n/a - d) No education, primary, secondary, third level vocational, university, unknown (%) = 0.2, 1.1, 52.9, 21, 24.7, 0.2 - e) n/a   f) n/a  g) N= 656 | Screening + BI did not involve provision of written information, advice, or recommendations of any sort  BI content: asking the woman about her drinking pattern; attitudes towards drinking; and any previous attempts to change drinking habits, including strategies used in the past.  a) RA; b) 1; c) 5 mins.; d) 32 wks. of gestation | - Screening + treatment as usual with no specific intervention in relation to AUP | - No significant difference in the mean AUDIT scores b/w the groups. | - n/a |
| (8) Tzilos et al., 2011;  USA, Providence, RI;  Prenatal clinic;  RCT | a) PW who drink alcohol  Alcohol, quantity (SD) (g of ethanol/ week): C= 83.4 (147), I= 89.5 (91.3)  b) M= 25 wks. | - (I, C) - a) - b) African-American; Caucasian, Others (%): I= 78, 19, 3; C= 87, 13, 0 - c) n/a - d) Grades 0-8, 9-11, high school graduate, college (%): I= 7, 44, 33, 15; - C: 13, 52, 26, 9 - e) 26% single (in both groups) - f) n/a - g) N= 50 | Screening + MI educational feedback based on participants’ motivation to quit or reduce AU, and relapse prevention   - MI framework: SDT sought to facilitate self-change and/or treatment-seeking, Decisional balance, feedback, and optional goal setting   Computer-delivered content: Importance of reducing AU during pregnancy. Content tailored based on drinking status of the participant. Delivered current information about FASD. All images and examples in the software were specifically tailored to PW.  a) Computerized; b) 1; c) 15-20 mins.; d) 1-month post-intervention. | - Screening + Received unrelated questionnaire + educational brochure on reduction of AUP | - No effect of treatment on Percentage using alcohol at FU (difference b/w the two groups) (P=0.71)- No statistics provided. | M head circumference: NS difference b/w groups (P=0.72)   - **M BW significantly more in BI v/s C (F(1, 44)=0.13), P=0.03) |
| (9) Wernette et al., 2018;  USA, Michigan;  Hospital;  RCT | a) PW, mostly low-income with SAU  b) M(SD)= = 13 wks. | - (I, C) - a) M(SD): I= 25.1(5.79); C= 23.2(4.30) - b) African-American, Caucasian, Native American/ Alaskan, More than 1 race, Other/unknown (%): I= 35, 23, 6, 12, 23; C= 10, 42, 0, 16, 32 - c) n/a - d) Grades 0-8, 9-11, high school, some college (%): I= 23, 23, 26, 39; C= 0, 42, 32, 26 - e) Single (%): I= 52; C= 42 - f) Full-time, part-time, student, home-maker, unemployed (%)= I= 23, 20, 6, 3, 48; C= 22, 10, 5, 26, 37   g) N= 50 | Computerized screening + (IMB) model based MI.  MI involved empathetic animated narrator education regarding health risks and included testimonial videos and pictures tailored plan with goal setting) + booster session: the animated narrator reviewed the personalized plan and identified barriers to increase safe behaviors + received brochures specifically designed to facilitate health risk behaviors during pregnancy.   - a) Computer; b) 2; c) 60 mins. + 15 mins. booster; d) 4-month FU post intervention | - Screening + Interaction with animated narrator at baseline and booster: watching brief segments of popular television shows with subsequent questions for ratings of their subjective preference + brochures specifically designed to facilitate health risky behaviors during pregnancy | - ** The odds of AU post-intervention is 84% lower in the intervention group v/s control group (OR= 0.16, 95%CI= 0.04 – 0.74) | - n/a |
| 1. (10) Rubio et al 2014; 2. USA, PA;   Urban Obstetrics Clinic;  RCT | a) PW who are risky drinkers. Alcohol dependence (%): C= 25.0, I= 22.1; Alcohol abuse (%): C= 24.2, I= 22.1  b) NMT 20 wks. | - (I,C) - a) M (SD): I=23.5(4.04); C=24.1(5.4 - b) Black, White, Others (%): I= 45.5, 51.5, 3; C= 40.6, 55.8, 3.6 - c) n/a - d) < high school, high-school or GED, some post-high school education, degree past high-school diploma: I= 18.8, 50, 22.9, 8.3; C= 25.5, 34.2, 28.2, 12.1 - e) Never married, presently married, divorced/ separated, other: Intervention (%): I= 63, 6.8, 19.9, 4.1, 6.2; C= 64.7, 11.3, 18, 4, 2 - f) Unemployed, home-maker, part-time student, full-time student, part-time worker, full-time worker (%): I= 8.2, 30.8, 4.8, 12.3, 18.5, 25.3; C= 12.2, 32.4, 2, 12.8, 11.5, 29.1 - g) N= 330 | Screening + MET- goal to motivate women to abstain from AU and encourage dependent women to accept referral to specialized treatment program- interview based on the participant’s ability to change their AU  MET FRAMES framework: AU, provided specific feedback based on use and alcohol risks to the fetus, and included a plan for changes in behavior, reinforce safe prenatal AU in women who had already eliminated alcohol, and encourage safe drinking behaviors after delivery to protect future pregnancies and to improve overall health  a) Trained registered nurse or lay counselor; b) 5; c) 10-30 mins.; d) post-enrolment: 4 and 8 wks.; 32 wks. of gestation; post-partum- 6 wks.; 6 months; and 12 months | - Screening + Usual care: standard warnings on AUP by prenatal clinic staff | - No significant difference in odds of AU post-partum (P=0.08), and drinks/ day after baseline (P=0.07) b/w the BI and control group. | - # BW significantly lower in those receiving BI compared to control (3014 grams vs 3160 grams; P = 0.04) |
| 1. (11) Van Der Wulp et al 2014;   Netherlands, Maastritch;  Midwifery Practices;  C-RCT | a) PW who drink  Binge drinkers (%): 1.2, Risky drinkers (T-ACE positive) (%)= 57.4  b) Max. 12 wks. Pregnant | - (T) - a) M (SD)= 32.56(4.20) - b) n/a - c) Low, Medium, High (%)= 11.3, 54.8, 33.9 - d) Low, Medium, High= 2.6, 31.2, 66.2 - e) n/a - f) Steady partner n(%)= 56.7 - g) N= 349 | 2 intervention arms:  screening + MI – focused on abstinence.  MI focal modality: I-Change model (Transtheoretical model + Theory of Planned Behavior   1. 1) Health counseling (HC): (Feedback 1) Step 1: Screening of participants and their partners, motivation to stop drinking; step 2: tailored advice based on participant’s goal   step 3: discussing barriers of abstinence and mobilization of social support; step 4: self-help guide, relevant websites, actions plan and coping strategies, access addiction services discussed; step 5: goal setting  Feedback session 2, step 6- reassessed AU and provided additional support to quit. Feedback session 3, step 7: discussed AU and its implications for breast-feeding  2) Computer Tailoring (CT): Feedback letter 1: same as health counseling; Feedback letter 2 (T1): personalized information on the respondents’ choice of characteristics assessed with the baseline questionnaire. Feedback letter 3: tailored to changes in the respondent characteristics assessed at T1  1) HC: a) midwife; b) 3-sessions (7 steps); c) Feedback-1: 10-15 mins.; Feedback2- 1 minute; Feedback 3: 1 minute; d) 3 and 6 months post-enrolment   - 2) CT: a) Computer; b) 3 sessions; c) n/a; d) 3 and 6 months post-enrolment | - Screening + Quick routine alcohol care in line with national guidelines- midwives recommend complete abstinence to the clients | - **CT v/s Control: A significant M of 0.43 less alcohol units/ week were consumed in the CT group compared to the Control: M (SD)= –0.43 (0.12) (95% CI= –0.67 to –0.18), P = .001. - HC v/s Control: Alcohol units consumed/ week difference: NS | - n/a? |
| 1. (12) Peles et al., 2014;   Israel, Tel Aviv;  Inpatient pre-delivery and emergency unit;  RCT | a) PW using alcohol or other substances (nicotine smoking, alcohol or drugs)  M(SD)= AUDIT scores: I= 5.4(3.9), C= 6.3(6.6); M(SD)= TWEAK scores: I= 0.3(1.7), C= 0.5(1)  b) M(SD, Range)=24.6 (6.3, 5-30) wks. | - (I, C) - a) M (SD): I= 32.6(4.7); C= 31.5(5.2) - b) n/a - c) n/a - d) ≥12 yrs (%): I= 48.5; C= 66.7 - e) Married/couple (%): I= 85.4; C= 95.5 - f) n/a - g) N= 210 | Screening + MI + newsletter  MI framework was FRAMES- mostly focused on reduction of other substances tobacco/ nicotine smoking besides drinking.  Education content: explained the dangers associated with AUP, and their grave implications for both PW and her baby  Newsletter: described the implications of exposure to these substances and how to avoid them; practical tips to help with the exposure reduction  a) RA; b) 1; c) N/A; d) Post-delivery common in both intervention and control | - Screening but only after their delivery | - NS group difference in M AUDIT scores. | - M APGAR1 min = intervention 8.7 (0.8) v/s control 8.1(2) P = 0.1. APGAR 5 min= intervention 9.5 (1.1) v/s control 9.5(1)P= 1., APGAR 1 and 5 minute scores: NS difference b/w groups |
| 1. (13) Osterman et al., 2012;   USA;  Prenatal clinics;  RCT | a) PW using alcohol  b) ≤36 wks. | - (T) - a) M=24.9 years - b) Non-Hispanic African American (%): 66.7 - c) Annual income of less than US$15,000 (%)= 64.3 - d) n/a - e) Single (%)= 83.0 - f) n/a - g) N= 171 | Screening + MI; in person: focused on decrease drinking.  MI to assist PW in reducing their AU  MI framework READS (establishing empathy, developing discrepancy, rolling with resistance, and supporting self-efficacy) + SDT   - a) Certified psychiatric mental health clinical nurse specialist; b) 1; c) 30 mins.; d) 4-6 wks. FU post-intervention | - Screening + Standard education on prenatal risk of alcohol | - M drinking days/ week (P=0.26); and M drinks/ day (P=0.85): difference b/w groups | - n/a |
| 1. (14) Marais et al., 2011;   SA;  Rural area clinics;  C-RCT | a) PW - drinkers  M(SD)= AUDIT scores: I= 6.9(8.6), C= 9.6(8.8)  b) <20 wks. | - (I,C) - a) M (SD): I= 24.3(6.3); C= 25.3(5.8) - b) Africans (%): I= 100; C= 68 - c) Schooling < 8 grade (%): I= 24; C= 20 - d) n/a - e) n/a - f) n/a - g) N= 194 | Screening + MI + summary booklet  MI: patient centred counseling strategy focusing on changing patient behavior and increasing patient compliance with therapy, were used in feedback sessions to negotiate and set goals; and to reinforce their behavior in FU sessions  a) Trained field workers; b) 3-4; c) 20 mins./ session; d) 1.5 months from baseline and just before birth | - Initial assessment interview included the consent form, the personal questionnaire, the AUDIT scores + AUP booklet | - **Significant reduction in M AUDIT scores in BI compared to control: M, (SD; P value) 1.97(0.64; 0.002) | - n/a |
| 1. (15) Yonkers et al., 2020;   USA;  Urban academic hospital-based clinics;  RCT | a) PW using alcohol and or other substances  (a score of 6 or more on ASSIST tool for PW- moderate risk)  b) n/a | - (NA; A) - a) M(SD) yrs: NA= 34 (11); A= 33 (11) - b) African-American, Hispanic-American, Caucasian-American, other (%): NA= 66, 15, 13, 5; A= 75, 13, 8, 4 - c) n/a   d) Some school or less, high school, beyond high school (%): NA= 35, 40, 26; A= 13, 74, 13  e) Married/ living with partner, other living situation: NA= 45, 55; A= 45, 55  f) Full-time, part-time, not working (%): NA= 15, 18, 67; A= 17, 33, 50  g) N= 439 | Screening + MI: motivation to quit or reduce AU.  MI framework: empathy, respect autonomy via open-ended questions, affirmations, reflections, and summaries, and goal changing, in part through techniques such as personalized feedback.  2 arms:  e-SBIRT: provided feedback; asked questions and provided text answers and images of items that could be earned with money saved from not buying substances.  SBIRT: discussion of the participant's SU and review of a handout listing local treatment resources  1) e-SBIRT- a) Computer; b) 1; c) 20 mins.; d) 1,3, and 6 months post-intervention   - 2) SBIRT: a) clinician; b) 1; c) 20 mins.; d) 1,3, and 6 months post-intervention | - EUC= Screening + told their level of risk and were given a pamphlet listing local treatments that could help them to cut down or stop their AU | - No treatment effect was observed for alcohol abstinence, however alcohol abstinence rates at 1 month, 3 month and 4 month follow-up were consistently higher among SBIRT (27%, 27, and 20%), compared to e-SBIRT (9%, 9%, and 13%), and EUC (0%, 8%, 0%) | - n/a |
| 1. (16) Joya et al., 2016;   Spain, Barcelona;  Hospital;  RCT | a) PW drinkers  b) First antenatal visit - after 1 month of gestation- mostly 2nd month | - (I, C) - a) M(SD): I=32.3(5.0); C = 29.9 (5.7) - b) Spanish (%): I= 36.8; C = 47 - c) n/a - d) Primary school, high school, university: I =3.6, 42.5, 21.2; C= 6.9, 48.2, 19.7 - e) n/a - f) Unskilled, skilled (%): I= 59.0, 18.5; C= 64.4, 16.8 - g) N= 168 | Screening + usual care + MI- change her risky behavior.  MI framework READS: establishing empathy, developing discrepancy, rolling with resistance, and supporting self-efficacy + SDT  MI content: personalized feedback of risk, motivate the woman to change target behaviors, decrease her temptation to engage in risk behavior and increase her confidence to avoid it, develop change plans, and encourage her to attend the contraceptive counseling visit + General health advice   - a) computerized; b) 1; c) N/A; d) post-partum | - Screening + General health advice for eating, drinking, health screenings, family planning, prevention of sexually transmitted infections and exercise recommended for women of childbearing age were transmitted to all the participants. | - NS group difference in Percentage of alcohol abstinence (P=0.285) | - n/a |
| 1. (17) Reynolds et al., 1995;   USA;  Public Health Clinics are mostly from urban setting and rural neighborhoods;  RCT | a) PW who drink. <25 wks. pregnant  M drinks/ month: I=44; C=28  b) n/a | (I, C)  a) M: I= 22.7; C= 22.2  b) African American, European (%): I= 61, 31; C= 64, 36  c) >$5,000 (%): I= 56; C= 61  d) n/a  e) n/a  f) n/a  g) N=78 | Screening + MI + self-help manual- focused on cessation + monitoring call after a week  MI framework SDT: goal setting, self-monitoring, perceived self-efficacy, negative outcome expectancies of drinking, positive outcome expectancies of cessation, and skills for cessation.  Education content: using 9 steps self-help manual (key ideas, skill building and exercises to quit alcohol) + a call after 1 week to check on the progress.  a) Educators; b) N/A; c) 10-15 mins.; d) 2 months post-enrolment | Screening + information on effects of AUP by the clinic + brief discussions with clinic staff + video tape on prenatal care | NS group difference in the alcohol quit rate (P=0.58) | n/a |
| 1. (18) Nilsen et al., 2010;   Sweden;  Maternity Care Center;  No randomization of participants to the intervention. Comparison of Cohort 1: registered for maternity care April 2006 - March 2006: Usual care v/s Cohort 2 April 2006-2007: Questionnaire based counseling | a) PW irrespective of their drinking status.  b)10-12 wks. of pregnancy | (I, C)  a) < 19, 20-24, 25-29; 30-34; 35-39; ≥40 yrs (%): I= 0.3, 6.6, 22.8, 45.5, 20.5, 4.3; C= 0, 5, 24.4, 46.3, 19.7, 4.6  b) n/a  c) n/a  d) Compulsory school, upper secondary, university/ college, other (%): I= 4.2, 28.5, 63.2, 4.3; C= 3, 30.9, 62.1, 4  e) n/a  f) n/a  g) N= 1855 | Screening + MI based on participants' AUDIT-C scores  Tailored: AUDIT-C score 0-5: oral and written information on consequences for the fetus if the woman drinks alcohol; 6-9: new appointment within the next 2–3 wks. to FU whether the woman has been able to abstain and to discuss strategies to refrain from drinking and book further appointments as required; 10 - 12: book an appointment with a physician at the maternity care center  a) Midwife; b) 1 + (more appointments given if required); c) 60-90 mins.; d) 30-32 wks. pregnancy | Screening + Midwife 60-minute-long: Screening. Regardless of the amount of alcohol consumption, the woman is recommended to abstain from AUP.  2^nd^ meeting in 30–32 gestation wks.: reassessment of AU | NS group difference in percentage abstinent (P = 0.92) | n/a |
| 1. (19) Sarvela et al., 1993;   USA, IL;  Regional health centers;  Non-equivalent control group: to avoid contamination bias- they systematically made sure that the people from intervention and control group were not from the same geographic locations | 1. Adolescent-PW using alcohol or other substances 2. n/a | n/a | Usual care + ASPEN (Self-administered educational modules in private + brief, private, q and a session)- following health communication  The eight modules: Cigarette Smoking and Your Unborn Baby; Your Baby’s Growth During Pregnancy; You, Your Baby, and Alcohol; Check with Your Doctor (use of OTC drugs); Smoking Marijuana and How it Affects Your Baby; Health and Nutrition; Stress and You; and Decision-Making.  a) Trained health worker; b) 1; c) n/a; d) n/a | Screening + Usual care - no information provided | *significant drop in alcohol use from baseline to post-intervention in BI (from 22.3% to 2.4%) and in control (from 13.1% to 1.7%). No between-groups comparison was provided | M APGAR scores provided but no P-values provided |
| 1. (20) Waterson et al., 1990;   UK, London;  Antenatal clinic-hospital;  (may be a c-RCT- 4 booking clinics/week that book patients randomly at the same hospital- 2 clinics allocated to each intervention and control group) | 1. PW who drink 2. n/a | g) N= 977 | Screening + BA+ written information to reduce alcohol intake to only 1 unit a day or 7 units a week.  Written information: not much information provided on the content of this leaflet  a) Doctors; b) 1; c) N/A; d) 28th week of pregnancy and in the week immediately prior to delivery | Screening + written information (no details provided on the content) | NS group difference in the percentage of women consuming alcohol | n/a |
| 1. (21) Yonkers et al., 2012;   USA, CT;  Hospital;  RCT | a) PW who use alcohol and other substances  MINI Drug or alcohol abuse or dependence= 28%  b) 1–8 wks.= 5%, 9–16 wks.= 32%, 17–24 wks.= 35%, 25 wks.or later: 29% | - (C, I, T) - a) < 20; 20-34; ≥35 yrs (%): 15, 20,17; 76, 74, 75; 9, 6, 8 - b) White; Black; Hispanic; Other (%): 20, 23, 22; 55, 51, 53; 20, 23, 23; 1, 2, 2 - c) n/a - d) < 12; 12; 13-15; ≥16 yrs of edu. (%) = 30, 38, 34; 36, 43, 40; 27, 17, 22; 7, 1, 4 - e) n/a - f) n/a - g) N= 183 | Screening + MET-CBT (Motivational enhancement, functional analysis, safe sexual behavior, communication, alcohol relapse prevention and problem-solving)  a) Research Nurse Therapist; b) 6; c) 30 mins. / session; d) at delivery, 3 and 6 months post-delivery | - Screening + Brief advice: manual provided guidance on the risks of SU, the importance of abstinence, and the benefit of seeking drug and alcohol treatment outside of the prenatal setting. Typically lasted around 1 minute | - NS group difference in the alcohol quit rate (P=0.58) | a) Preterm birth: NS difference b/w groups (P=0.08)  b) LBW: NS difference b/w groups (P=0.41) |
| 1. (22) Meberg et al., 1986; 2. Norway, Tmsberg;   Midwife’s office- for intervention  Obstetrics department- control;  Quasi experimental- time cohort (retrospective estimation of AU during pregnancy) | 1. PW irrespective of their drinking status 2. n/a | (I, C)  a) M yrs: I= 26.9; C= 26.6  e) Married or living with  father of the baby (%): I= 95; C= 91  f) Employed, housewife (%): I= 52, 45; C=43, 54  g) N= 74 | Screening + Supportive counseling focused on reduction of alcohol consumption and its benefits to the fetus. Guilt-provoking criticism was avoided.  a) Midwife; b) 6; c) 60 mins. / session; d) N/A | Screening only at FU | No comparison b/w groups provided. However, significant post- intervention increases in percentage abstinent observed in both groups compared to baseline | n/a |
| (23) Winhusen et al., 2008  USA, NC;  12- CTP Clinics;  RCT | a) PW with SAU  b) M= 20 wks. | - (I, C) - a) M(SD): I = 27.3(5.8); C= 25.1 (4.8) - b) African-American, Caucasian, Hispanic, Others (%): I= 54.4, 44.1, 1.5, 0.0; C= 39.7, 56.9, 1.7, 1.7 - c) n/a   d) M(SD) education yrs: I= 11.2(1.8); C= 11.7(1.5)  e) Married, Separated/divorced, Not married (%): I= 14.9, 18.9, 66.2; C= 1.6, 18.0, 80.3  f) Employed Full/Part time (%): I=20.3; C= 29.5  g) N=200 | Screening + The MET-PS  MET-PS:  1^st^ session: report building and exploring participant’s feelings on her pregnancy, and perceived pros and cons of using substances, including AEP on their fetus.  2^nd^ session: individualized feedback and promoting activities for healthy pregnancy  3^rd^ session: tailored plan and support based on participants’ ability to change  a) Clinician administered the intervention and RA for FU; b) 3; c) 1^st^ session: 1.5-2 hours, 2^nd^ session and 3^rd^ session: approximately 60 mins.; d) 2 and 4 months post-intervention | At least 3 individual sessions with a clinician lasting for the same time as the intervention.  MET-PS and TAU sessions could be distinguished based on 10 items each were used to assess the clinician’s utilization of MET (e.g., avoiding confrontation, asking open-ended questions, reflective listening) general substance abuse counseling (e.g., SU assessment, discussion of SUP, treatment planning), and strategies inconsistent with MET (Anti-MET; e.g., confrontation of denial, asserting the authority of the therapist). | - NS difference b/w groups for M days of drug or AU during the entire 4-month period observed: Treatment (Z=1.11, P>.05), Treatment × Week (Z=−0.95, P>.05), Week (Z=0.02, P>.05), or Site × Treatment × Week (X2=0.68, df=2, P>.05) effects | - n/a |
| 1. (24) Chang et al., 2005;   US, Boston, MA;  Hospital-based prenatal clinic;  RCT | a) PW at-risk for drinking  All participants are T-ACE positive  b) Median (IQR)= 21(17-27) wks. | (I, C)  a) Median: I=32, C=30.7  b) African American, White, Others (%): I=8.6, 78.4, 13, C= 6.6, 78.8, 14.6%  c) Median income (by zip code): I= 54,676$; C= 55,700$  d) Median yrs of education: I= 16; C= 16.3  e) Married/ Partner (%): I= 82.2; C= 78.8  f) n/a  g) N=204 | Screening + Enhanced BI (MI/CBT) involving a) PW’s partners - focused on alcohol abstinence.  1) Healthy pregnancy facts knowledge assessment with feedback, 2) contracting and goal setting, 3) behavioral modification, and 4) summary.  a) Clinicians with at least Master's degree; b) 1; c) 25 mins. (average); d) prenatal | Screening by RAs: pregnant participants completed the 1) TLFB: to obtain estimates of their daily drinking for the 6 months before study enrollment 2) Alcohol Abstinence Self-Efficacy scale: evaluations of their perceived temptation to drink and their efficacy to abstain in 20 common situations and 3) Healthy Pregnancy Facts, a series of 7 statements about healthy habits during pregnancy that the respondent was asked to judge as true or false. | **Percentage of drinking days were significantly lower in BI compared to the controls (b=-0.163 (0.063); P<0.01) | n/a |
| (25) Moura et al., 2019 ;  Brazil, Sao Carlos, Ibate ;  Women Health Reference Center;  RCT | a) PW user of alcohol and/ or tobacco  M(SD) AUDIT score- C = 18.9 (8.0), I= 3.38 (2.97); M(SD)= T-ACE: C= 1.85 (1.144), I= 1.63 (0.744)  b) n/a | - g) N=24 | Screening + home-based CBT using educational booklet + 2 weekly monitoring calls for 2 wks.  CBT framework: “5R strategy” (relevance, risks, rewards, resistance and repetition.  Education booklet content: RA read the booklet aiming to increase the degree of motivation of PW to cease AUP and explaining FAS; included motivational phrases promoting self-efficacy  a) RA; b) 4; c) 7 mins. / session; d) 3 wks. and 2 wks. post-intervention | - Same as the intervention group, but no monitoring calls | - No comparison b/w groups provided. However, significant post- intervention reduction in M AUDIT scores and T-ACE scores observed in both groups compared to baseline | - n/a |
| (26) De Vries et al., 2015;  SA, Western Cape;  Community Health Clinics;  Pre-post study design | a) PW heavy drinkers  M(SD) AUDIT score= 18.9 (8.0)  b) n/a | (T)  a) M(SD)= 24.9 (5.4)  b) n/a  c) n/a  d) n/a  e) n/a  f) Full-time, Part-time, Seasonal, Unemployed (%)= 28.8, 6.1, 10.6, 54.5  g) N= 67 | Screening + MI+ CRA encourage positive changes in lifestyle/ drinking, childbearing practices  MI + CRA principles: expressing empathy through reflective listening; developing discrepancy in clients about negative impact of current behavior on important goals and values; rolling with resistance, supporting self-efficacy  a) Field staff | No control group | Weekend alcohol consumption drops significantly post-intervention in both 2^nd^ and 3^rd^ trimester compared to baseline (P<0.001) | n/a |

*AEP= Alcohol Exposed Pregnancy; APGAR= Appearance, Pulse, Grimace, Activity, and Respiration; AU= Alcohol Use; AUDIT= Alcohol use disorders identification test; AUDIT-C= Alcohol use disorders identification test- consumption; AUP= Alcohol Use in Pregnancy; BA= Brief Advice; BDP= Brief Drinker Profile; BI= Brief Interview; BW= Birth Weight; b/w= between; CA= California; CBT= Cognitive Behaviour Therapy; CRA= Community Reinforcement Approach; C-RCT= Cluster Randomized Control Trials; C= Control; CT= Computer Tailored; CTP= Community Treatment Program sites; EEC= Enhanced Education Control ES= Early Start ; ESP= Early Start Plus; EUC= Enhance Usual Care; FASD= Fetal Alcohol Spectrum Disorders; FRAMES = Feedback, Responsibility, Advice, Menu of the options, Empathy, Self-efficacy; HC= Health Counseling in-person; I= Intervention; IL= Illinois; IMB= Information-Motivation-Behavior model; IQR= Intra quantile range; LA= Los Angeles; LBW= Low Birth Weight; MA= Massachusetts; max. = maximum;* *M= Mean; MET= Motivational Enhancement Therapy; MET-CBT= Motivational Enhancement Treatment – Cognitive Behavioural Therapy; MET-PS= Motivational Enhancement Therapy for Pregnant substance users; MI= Motivational Interview; mins= minutes; MIS= Mississippi; n/a = information not available; NC= North Carolina; NICU= Neonatal Intensive Care Unit; NM= New Mexico; NS= non- significant; OC= Orange County; OH= Ohio; OR= Odds Ratio; P- value / P= probability value; PA= Pennsylvania; PW= Pregnant Women; READS= establishing empathy, developing discrepancy, rolling with resistance, and supporting self-efficacy; RI= Rhodes Island; RR= Risk Ratio; RCT= Randomized Control Trials; SA= South Africa; SAUP= Other-substance and alcohol use in pregnancy; SD= standard deviation ; SU= Substance Use; SUP= Substance use in Pregnancy; T= total participants; T-ACE= Tolerance, Annoyed, Cut Down, Eye-opener; TLFB= Alcohol Timeline Follow back; TWEAK= Tolerance Worry about drinking, Eye Opener, Amnesia, cut down on drinking; USA= United Sates of America; UK= United Kingdom; v/s = versus; wks.= weeks; yrs= years; 95% CI= 95% Confidence Interval;** Significant treatment effect favouring brief intervention; ## Significant treatment effect favouring the control condition; * Small-moderate treatment effect favouring brief intervention.*

References

1. Armstrong MA, Kaskutas LA, Witbrodt J, Taillac CJ, Hung Y-Y, Osejo VM, et al. Using drink size to talk about drinking during pregnancy: a randomized clinical trial of Early Start Plus. Soc Work Health Care. 2009;48(1):90-103.

2. Chang GW-H, Louise;Berman, Susan;Goetz, Margaret Ann. Brief intervention for alcohol use in pregnancy: A randomized trial. [References]: Addiction. Vol.94(10), 1999, pp. 1499-1508.; 1999.

3. Handmaker NS, Miller WR, Manicke M. Findings of a pilot study of motivational interviewing with pregnant drinkers. J Stud Alcohol. 1999;60(2):285-7.

4. O'Connor MJW, Shannon E. Brief intervention for alcohol use by pregnant women. [References]: American Journal of Public Health. Vol.97(2), 2007, pp. 252-258.; 2007.

5. Ondersma SJ, Beatty JR, Svikis DS, Strickler RC, Tzilos GK, Chang G, et al. Computer-Delivered Screening and Brief Intervention for Alcohol Use in Pregnancy: A Pilot Randomized Trial. Alcohol Clin Exp Res. 2015;39(7):1219-26.

6. Osterman RL, Carle AC, Ammerman RT, Gates D. Single-session motivational intervention to decrease alcohol use during pregnancy. J Subst Abuse Treat. 2014;47(1):10-9.

7. Sheehan J, Gill A, Kelly BD. The effectiveness of a brief intervention to reduce alcohol consumption in pregnancy: a controlled trial. Ir J Psychol Med. 2014;31(3):175-89.

8. Tzilos GK, Sokol RJ, Ondersma SJ. A randomized phase I trial of a brief computer-delivered intervention for alcohol use during pregnancy. J Womens Health (Larchmt). 2011;20(10):1517-24.

9. Tzilos Wernette G, Plegue M, Kahler CW, Sen A, Zlotnick C. A Pilot Randomized Controlled Trial of a Computer-Delivered Brief Intervention for Substance Use and Risky Sex During Pregnancy. J Womens Health (Larchmt). 2018;27(1):83-92.

10. Rubio DM, Day NL, Conigliaro J, Hanusa BH, Larkby C, McNeil M, et al. Brief motivational enhancement intervention to prevent or reduce postpartum alcohol use: a single-blinded, randomized controlled effectiveness trial. J Subst Abuse Treat. 2014;46(3):382-9.

11. van der Wulp NY, Hoving C, Eijmael K, Candel MJ, van Dalen W, De Vries H. Reducing alcohol use during pregnancy via health counseling by midwives and internet-based computer-tailored feedback: a cluster randomized trial. J Med Internet Res. 2014;16(12):e274.

12. Peles E, Sason A, Bloch M, Maslovitz S, Dollberg S, Many A, et al. The Prevalence of Alcohol, Substance and Cigarettes Exposure among Pregnant Women within a General Hospital and the Compliance to Brief Intervention for Exposure Reduction. Isr J Psychiatry Relat Sci. 2014;51(4):248-56.

13. Osterman RL, Dyehouse J. Effects of a motivational interviewing intervention to decrease prenatal alcohol use. West J Nurs Res. 2012;34(4):434-54.

14. Marais S, Jordaan E, Viljoen D, Olivier L, de Waal J, Poole C. The effect of brief interventions on the drinking behaviour of pregnant women in a high‐risk rural South African community: a cluster randomised trial. Early Child Development and Care. 2011;181(4):463-74.

15. Yonkers KA, Dailey JI, Gilstad-Hayden K, Ondersma SJ, Forray A, Olmstead TA, et al. Abstinence outcomes among women in reproductive health centers administered clinician or electronic brief interventions. J Subst Abuse Treat. 2020;113:107995.

16. Joya X, Mazarico E, Ramis J, Pacifici R, Salat-Batlle J, Mortali C, et al. Segmental hair analysis to assess effectiveness of single-session motivational intervention to stop ethanol use during pregnancy. Drug Alcohol Depend. 2016;158:45-51.

17. Reynolds KD, Coombs DW, Lowe JB, Peterson PL, Gayoso E. Evaluation of a Self-Help Program to Reduce Alcohol Consumption among Pregnant Women. International Journal of the Addictions. 1995;30(4):427-43.

18. Nilsen P, Holmqvist M, Bendtsen P, Hultgren E, Cedergren M. Is questionnaire-based alcohol counseling more effective for pregnant women than standard maternity care? J Womens Health (Larchmt). 2010;19(1):161-7.

19. Sarvela PD, Ford TD. An evaluation of a substance abuse education program for Mississippi delta pregnant adolescents. J Sch Health. 1993;63(3):147-52.

20. Waterson EJ, Murray-Lyon I. Preventing fetal alcohol effects; a trial of three methods of giving information in the antenatal clinic. Health Education Research. 1990;5:53-61.

21. Yonkers KA, Forray A, Howell HB, Gotman N, Kershaw T, Rounsaville BJ, et al. Motivational enhancement therapy coupled with cognitive behavioral therapy versus brief advice: a randomized trial for treatment of hazardous substance use in pregnancy and after delivery. Gen Hosp Psychiatry. 2012;34(5):439-49.

22. Meberg A, Halvorsen B, Holter B, Ek IJ, Askeland A, Gaaserud W, et al. Moderate alcohol consumption—need for intervention programs in pregnancy? Acta Obstetricia et Gynecologica Scandinavica. 1986;65(8):861-4.

23. Winhusen T, Kropp F, Babcock D, Hague D, Erickson SJ, Renz C, et al. Motivational enhancement therapy to improve treatment utilization and outcome in pregnant substance users. J Subst Abuse Treat. 2008;35(2):161-73.

24. Chang G, McNamara TK, Orav EJ, Koby D, Lavigne A, Ludman B, et al. Brief intervention for prenatal alcohol use: a randomized trial. Obstet Gynecol. 2005;105(5 Pt 1):991-8.

25. MOURA AAMD. EFEITO DO MONITORAMENTO POR TELEFONE DE INTERVENÇÕES BREVES PARA USO DE ÁLCOOL E TABACO ENTRE GESTANTES: ensaio clínico randomizado: UNIVERSIDADE FEDERAL DE SÃO CARLOS

CENTRO DE CIÊNCIAS BIOLÓGICAS E DA SAÚDE; 2019.

26. de Vries MM, Joubert B, Cloete M, Roux S, Baca BA, Hasken JM, et al. Indicated Prevention of Fetal Alcohol Spectrum Disorders in South Africa: Effectiveness of Case Management. Int J Environ Res Public Health. 2015;13(1):ijerph13010076-ijerph.
