# Supplementary material for: Effectiveness of brief alcohol interventions for pregnant women: a systematic literature review and meta-analysis
Source: BMC Pregnancy Childbirth. 2023 Jan 24;23:61. doi: 10.1186/s12884-023-05344-8 (PMC9872314; doi:10.1186/s12884-023-05344-8)
Supplement: Supplementary file 4 — Additional file 4. Screening tools and outcome measures. [file 12884_2023_5344_MOESM4_ESM.docx]

**Additional File 4. Screening tools and outcome measures**

| **Reference** | **Alcohol use screening tools pre- and post-intervention; important cut-offs for classifying alcohol users** | **Outcome measures (OR, RR; Mean difference etc.)** |
| --- | --- | --- |
| Armstrong et al.  (2009) | ES screening questionnaire (combination of CAGE and TWEAK screening tool) | Neonatal outcomes only:   - OR Low birth weight; OR preterm delivery - OR NICU admission - (Maternal alcohol use reduction not an outcome of interest) |
| Chang et al.  (2005) | - T-ACE; NIAAA quantity; Alcohol TLFB; Alcohol Abstinence self-efficacy scale - T-ACE positive= total score of ≥2. Risk of alcohol exposure= any alcohol consumption in the 3 months before study enrollment (while pregnant), or consumption of at least one drink per day in the 6 months before study enrollment, or drinking during a previous pregnancy - frequency questions | Beta-coefficient (SE):   - Drinks/ drinking days (quantity) - % of drinking days (frequency) |
| Chang et al.  (1999) | - Structured Clinical Interview for DSM-III-R to generate standard diagnoses; ASI; T-ACE; AUDIT; SMAST; TLFB - Risky drinking: T-ACE tolerance question was positive (and scored two points) if the women reported to feel high with two drinks or more of alcohol, rather than more than two drinks. | - Mean reduction in drinks/ drinking days from baseline to delivery; - Mean number of drinking episodes - Risk Ratio for any alcohol consumption during pregnancy - % of those maintaining their abstinence from pre-assessment to follow-up. |
| Handmaker et al.  (1999) | BDP; FDP  All drinking was converted into standard ethanol content (SEC) units equal to 0.5 oz (15 ml) of absolute alcohol. Light-moderate drinkers: < 1 SEC daily; Heavy drinkers=>/=1 SEC daily. | - Total SECs (reduction in the total drinks consumed) - Estimated peak blood alcohol concentration (BAC) - Total days abstinence during the most recent 2 months of pregnancy - % reporting total abstinence at follow-up |
| O'Connor & Whaley  (2007) | - TWEAK-5 (for heavy-risk drinking assessment); (MAX (valid predictor for teratogenic effect scored 2 or higher on the TWEAK scale, which has been suggested as a cut point for pregnant women who may not be alcohol dependent but who may, nevertheless, drink at levels that place the fetus at risk) | - Mean maximum drinks/drinking occasion (beta coefficient) - OR of being abstinent - OR of MAX |
| Ondersma et al.  (2015) | - T-ACE; TLFB | - % and OR in past 90-day period prevalence of abstinence, drinking days, drinking days with ≥3 drinks - Neonatal outcome:   % and OR of Healthy pregnancy (live birth of ≥2,50 grams with no admission to neonatal intensive care unit) |
| Osterman et al.  (2014) | AUDIT-10; QDS; TLFB | - Average number of drink days/ week - Drinks per day in the last 30 days |
| Sheehan et al.  (2014) | AUDIT-  Binge drinking=5 or more units of alcohol/ occasion | - Mean AUDIT scores (SD) |
| Tzilos et al.  (2011) | - T-ACE; TLFB- modified computer version | - % of abstinence - Neonatal outcomes - Mean birth weight - Difference in mean head circumference - Difference in mean birth weight |
| Wernette et al.  (2018) | - A positive score on T-ACE (screening tool for at-risk drinking); SURP-P; TLFB | - OR of alcohol use |
| Rubio et al.  (2014) | Alcohol use: validated tool by Maternal Health Practices and Child Development Project.  Alcohol use disorder: CIDI (diagnosis based on ICD10 and 4th DSM-1V)  Drinking episode: alcohol use at least weekly before the pregnancy and/or reported any **binge** of ≥3 drinks on one occasion during the year before pregnancy  Hazardous drinking:>1 drink/day on an average | A) Neonatal outcomes: head circumference; body weight; body length. B)Drinks/ day; c) % of any alcohol use post-partum. *Change in pre-natal alcohol use not analysed due to limited data. C) OR: any alcohol use; b-coeffecient: drinks/day after baseline |
| Van Der Wulp et al.  (2014) | 5-item Dutch QFV; T-ACE  Binge drinking: if respondents had ≥3 standard glasses of alcohol (10 gms. of pure alcohol) on 1 day since they knew they were pregnant  Risky drinking=used T-ACE drinking tool (0=non-risky and 1-risky). | - % of alcohol abstainers; Average standard drinks/week. |
| Peles et al.  (2014) | AUDIT-10 for hazardous and risky drinking; AUDITC- for alcohol misuse. Alcohol misuse: ≥3 drinks/ sitting. TWEAK (for risky drinking during pregnancy); ASI | - Neonatal: birthweight; gestational age; APGAR 1and 5 minutes. Alcohol use: Mean AUDIT and TWEAK score |
| Osterman et al.  (2012) | AUDIT-10 | - Previous 30 day: a) Mean number of drinking days in a typical week (SD); b) Mean number of standard drinks in a typical day |
| Marais et al.  (2011) | AUDIT | - Mean AUDIT (SD); Intervention effect |
| Reynolds et al.  (1995) | 25 item questionnaire to identify women who  drank in the past month; TACE for problem drinking. Quit drinking: stopped all four of measured alcohol beverages (beer, wine, liquor, mixed drinks). Pre-test and post-test questions were similar with some changes | - Alcohol abstinence/ quit rate; Total amount of alcohol consumed past month (number of days drinking for each beverage by the average number of drinks consumed per occasion) |
| Waterson et al.  (1990) | Questionnaires - name unspecified | - % at daily safe level of alcohol; % drinking <7 units of alcohol/week when planning pregnancy and during pregnancy |
| Nielsen et al.  (2010) | AUDIT-C | - % who ceased drinking when pregnancy was recognised and during pregnancy |
| Sarvela et al.  (1993) | N/A | - % using alcohol in the past five month; Mean APGAR score |
| Yonkers et al.  (2020) | ASSIST; TLFB- all substances use | - % abstinent |
| Joya et al.  (2016) | AUDIT; TLFB | - % abstinent (ETG<7 pg/mg); % moderate consumers (ETG between 7-30 pg/mg); Percentage high consumers (ETG > 30 pg/mg) |
| Yonkers et al.  (2012) | TWEAK; modified TWEAK; TLFB (past 28 days) | - Neonatal outcomes: % of preterm birth (birth of less than 37 completed weeks gestation); % of low birth weight (weight less than 2500 grams). Alcohol use: Mean number of days for drug/ alcohol use; % abstainers from both drug and alcohol (based on self-report and urine test) |
| Meberg et al.  (1986) | Cahalan method: grams of absolute alcohol per day | - % teetotalers |
| Winhusen et al.  (2008) | TLFB | - Mean days of drug/ alcohol use per month |
| Moura et al.  (2019) | T-ACE; AUDIT-C; ASSIST  A result less than two in the T-ACE is characterized as low risk and greater than 2 points is understood as risk consumption. A score of ≥3 on the AUDIT-C must be carefully assessed for preventive interventions and, considering pregnancy, the cut-off for AUDIT C is zero, since no amount of alcohol is safe. | - % abstinence; Mean AUDIT-C; Mean T-ACE |
| De Veris et al.  (2015) | AUDIT | - Mean total weekend drinks |
| Xu et al.  (2017) | Modified TWEAK, ASI | - % of days of alcohol/ any drug use in the past 28 days-not reported just mentioned that there was no treatment effect. Cost-minimization: intervention costs, hospital facility costs, physician fees, and costs of psychotropic medications from baseline to 3 months post-partum. |

*AUDIT= Alcohol use disorders identification test; AUDIT-C= Alcohol use disorders identification test- consumption; ASI= Addiction Severity Index; Brief drinker profile= BDP; CIDI= Composite International Diagnostic Interview (diagnosis based on ICD10 and 4th DSM-1V); ES= Early Start; FDP= Follow-up drinker profile; MAX= Maximum drinking per occasion; max. = maximum; n/a= no information available; NIAAA= National Institute on Alcohol Abuse and Alcoholism; QDS= Quick drinking Screen; QFV= Quantity-Frequency-Variability; SMAST= Short Michigan Alcohol Screening Tests; SURP-P= Substance Use Risk Profile Pregnancy scale; T-ACE= Tolerance, Annoyed, Cut down, Eye opener; TLFB= Timeline Follow Back; TWEAK= Tolerance Worry about drinking, Eye Opener, Amnesia, cut down on drinking; %= Percentage*
