# Supplementary material for: Effectiveness of brief alcohol interventions for pregnant women: a systematic literature review and meta-analysis
Source: BMC Pregnancy Childbirth. 2023 Jan 24;23:61. doi: 10.1186/s12884-023-05344-8 (PMC9872314; doi:10.1186/s12884-023-05344-8)
Supplement: Supplementary file 5 — Additional file 5. Quality assessment of studies included in the systematic review. [file 12884_2023_5344_MOESM5_ESM.docx]

**Additional File 5. Quality assessment of studies included in the systematic review

Table 1. Quality assessment of RCTs using the Centre for Evidence-based Medicine (CEBM) Oxford critical appraisal tool for RCTs (1)**

| **Reference** | **What questions did the study ask? PICO (are the results of the trial valid?)** | **Was the assignment of patients to treatments randomized?** | **Were the groups similar at baseline?** | **Aside from the allocated treatment, were groups treated equally?** | **Were all patients who entered the trial accounted for? Were they analyzed in the groups to which they were randomized?** | **Were measures objective or were the patients and clinicians kept "blind" to which treatment was being received?** | - **What were the results? How large was the treatment effect?** | **What is the measure? What does it Mean?** | **How precise was the estimate of the treatment effect?** |
| --- | --- | --- | --- | --- | --- | --- | --- | --- | --- |
| (2) Chang et al  2005 | Y | Y | U | Y | Y, U | N  Note: RA at FU blinded | Beta-coefficient (SE): Drinks/ drinking days=-0.016(0.042); Percentage of drinking days=-0.163 (0.063). | Beta-coeffecient (SE). Percentage of drinking days were significantly lower in BI compared to the controls (b=-0.163; P<0.01)- BI more effective than the control | Point estimate; SE and P-value provided |
| (3) Chang et al  1999 | Y | Y | Y | Y | U, U | N  Note: RA at FU blinded | Risk Ratio for alcohol consumption during pregnancy (BI v/s control) = 0.80; P=0.33. Neonatal outcome: Birth weight (BI v/s control) = 3360 v/s 3406 grams. APGAR-1 = (8.1 v/s 7.8). APGAR-5= (8.9 v/s 8.7) | The risk for AU post-intervention is NS different in BI compared to control and APGAR - 1 and 5, and BW | Point estimate; SE; and P-value provided for AU outcomes. Point estimate only provided for neonatal outcomes- no SE or 95%ci OR P-value provided- only mentioned that the differences b/w groups are insignificant in the text. |
| (4) Handmaker et al  1999 | Y | Y | Y | Y | U, U | N  Note: RA blinded at FU | Effect size θ - (standardized M difference b/w two populations): within group difference is comparable b/w BI (θ=0.40; 0.46; 0.20) and control (θ=0.46, 0.77, 0.69), for changes in consumption, BAC, and abstinence, respectively. The effect ranges from small to medium. | The standardized difference in BI v/s control group for the M drinks consumed, changes in the percentage of abstainers, and estimated peak BAC is comparable. | Point estimate and p-value given; no 95% CI provided P-value provided |
| (5) O'Connor & Whaley et al  2007 | Y | Y | Y | Y | N, Y | N | OR: abstinent (OR=5.39; 95% CI = 1.59, 18.25) | There is 5.39 times higher odds of being abstinent if received a BI compared to control | Both point estimate and 95% CI provided |
| (6) Moura et al  2019 | Y | Y | U | Y | Y, U | N | Y, U | No comparison b/w groups provided. However, significant post- intervention reduction in M AUDIT scores and T-ACE scores observed in both groups compared to baseline | Point estimates, SD and P-vales provided |
| (7) Ondersma et al 2015 | Y | Y | Y | Y | Y, Y | N | OR: 90-day period prevalence abstinence= aOR: 3.4 (0.5-21.0) (aOR is adjusted for baseline AU). Healthy pregnancy aOR=3.3(0.8 to 13.8) | The odds of 90-day period and healthy pregnancy prevalence abstinence b/w BI and control group were not significantly different | Point estimate and 95%CIs provided |
| (8) Osterman et al 2014 | Y | Y | Y | Y | Y, Y | N | **I:** 30 days post-baseline: Average drinks per week 0 (SD=0); Average of 0.05 drinks per day (SD=0.30); AUDIT scores = 0.45(0.98); C: 30 days post-baseline: Average drinks per week 0.04(SD=0.29); Average of drinks per day 0.04(0.29); AUDIT scores =0.35(1.03). | Drinking behaviors did not change significantly across time for drink days per week or drinks per day, nor did the groups generally differ from each other on these variables | No direct information on treatment effect. |
| (9) Sheehan et al 2014 | Y | N | N | Y | Y, Y | N | M AUDIT score difference pre-post (BI v/s control): No significant difference in the M score b/w the groups. | Participants in the two groups did not differ in terms of AUDIT score regarding their alcohol consumption post-intervention (time 3); M score in those who had received the brief intervention was 0.8 (standard deviation 1.3) and M score in the control group was 0.7 (standard deviation 1.3) (t = − 0.6, p = 0.520) | Point estimate, SD, and P-value provided |
| (10) Tzilos et al  2011 | Y | Y | Y | Y | Y, U | Y | Percentage of no drinking at follow-up; Neonatal outcomes: M birth weight; difference in M head circumference and birth weight | No effect of treatment on AU at follow-up (difference b/w the two groups) (p=0.71)- No statistics provided. Treatment group differences were not observed for gestational age (F(1,44)=01.97), p=0.17; or head circumference (F(1, 44)= 0.13), p=0.72). Significant differences in birthweight (in favor of intervention) (F(1, 44)=0.13), p=0.03). M birthweight of intervention group was 3189.6 (SD=328.0), v/s control which was a M of 2965.3 (SD=387.7; d=0.62) | Only P-value provided for AU. Point estimates, SD, and P-value provided for neonatal outcomes |
| (11) Wernette et al  2018 | Y | Y | Y | Y | Y, U | N | OR: BI v/s control (time by group interaction): reduction in odds of alcohol intake (0.16 (0.04, 0.74) | The odds of AU post-intervention is 84% lower in the intervention group v/s the control group | Yes, point estimate and 95% CI provided |
| (12) Rubio et al  2014 | Y | Y | Y | Y | Y, Y | N  Note: RA at FU blinded | OR: Any AU post partum=0.50; 95% CI 0.23–1.09; P = 0.08. B-coefficient (95%CI): drinks/day after baseline =−0.11; 95% CI−0.23–0.01; P = 0.07) | No significant difference in any AU post-partum and drinks/ day after baseline b/w the intervention and control group. | Point estimate; 95% CI and P-value provided |
| (13) Peles et al  2014 | Y | Y | N | Y | U, U | N | M alcohol AUDIT score life (0.4±0.9 among 9 of control  vs. 1.7±1.5 among 14 of the intervention, P=0.03). M APGAR1 min = intervention 8.7 (0.8) v/s control 8.1(2 ) P = 0.1. APGAR 5 min= intervention 9.5 (1.1) v/s control 9.5(1), P= 1 | No significant difference in M AUDIT, APGAR 1 and 5 minute scores b/w intervention and control | Point estimate, SD and P-value provided |
| (14) Osterman et al  2012 | Y | Y | Y | Y | Y, Y | U | Past 30 days: M drinking days/ week (1.09 intervention v/s 1.54 control, P=0.26); M drinks/ day = (2.38 intervention v/s 2.29 control, P=0.85) | No significant difference for AU b/w intervention and control | Point estimate and P value provided |
| (15) Reynolds et al  1995 | Y | Y | Y | Y | Y, U | N | Alcohol quit rate: 88% intervention v/s 69% control, P=0.58 | No significant difference in the quit rate b/w intervention and control group | Point estimate and P-value provided |
| (16) Waterson et al  1990 | Y | U | Y | Y | N, U | U | Only difference in percentage of safe drinkers no effect size given | Just mentioned as text: no significant differences in terms of the numbers of women consuming 1 unit or more a day either within or b/w trials. | Point estimate provided, P-values not given for all |
| (17) Nilsen et al  2010 | Y | N | Y | U | N, N | N | Percentage ceased drinking= (93.2% v/s 93.1%; P=0.92) | NS difference in the quit rate for intervention v/s standard care | Point estimate and P-value given |
| (18) Sarvela et al  1993 | N  Note: usual care in control no description provided | N | N | U | Y, Y | N | Only percentages of AUDIT pre-test and post-test provided and APGAR scores- no P-vale provided | Cannot make any interpretation | Point estimate only provided |
| (19) Yonkers et al  2020 | Y | Y | U | Y | Y, Y | U | NS | No significant difference in the percentage of abstinence b/w groups | Point estimate and P-value |
| (20) Joya et al  2016 | Y | Y | Y | Y | Y, Y | N  Note: Blinding at analysis level | NS group difference in Percentage of alcohol abstinence (P=0.285) | No significant difference in the BI v/s control in AU | Point estimate, P-value given |
| (21) Yonkers et al  2012 | Y | Y | U | Y | Y, Y | U | OR=Percent abstinent from both drugs and alcohol according to combined self-report and urine=MET-CBT vs. BA: intake to delivery 0.77 (0.32 to 1.84)  MET-CBT vs. BA: delivery to 3 months post-delivery 0.76 (0.27 to 2.11) | NS difference in the odds of percentage of alcohol abstinence b/w control and intervention | Point estimate, P-value and 95% CI given |
| (22) Winhusen et al 2008 | Y | Y | Y | N | Y, Y | N | M days of drug or AU during the entire 4-month period observed no significant Treatment (Z=1.11, P>.05), Treatment × Week (Z=−0.95, P>.05), Week (Z=0.02, P>.05), or Site × Treatment × Week (X2=0.68, df=2, P>.05) effects | BI and control groups are comparable | Point-estimate and P-values provided |
| (23) Meberg et al 1986 | Y | N | N | Y | N, U | N | No Information on effect size | n/a | No Information on effect size |

*N=no; Y= Yes; U= Unclear AEP= Alcohol Exposed Pregnancy; APGAR= Appearance, Pulse, Grimace, Activity, and Respiration; AU= Alcohol Use; AUDIT= Alcohol use disorders identification test; AUDIT-C= Alcohol use disorders identification test- consumption; AUP= Alcohol Use in Pregnancy; BA= Brief Advice; BDP= Brief Drinker Profile; BI= Brief Interview; BW= Birth Weight; b/w= between; CA= California; CBT= Cognitive Behaviour Therapy; CRA= Community Reinforcement Approach; C-RCT= Cluster Randomized Control Trials; C= Control; CT= Computer Tailored; CTP= Community Treatment Program sites; EEC= Enhanced Education Control ES= Early Start ; ESP= Early Start Plus; EUC= Enhance Usual Care; FASD= Fetal Alcohol Spectrum Disorders; FRAMES = Feedback, Responsibility, Advice, Menu of the options, Empathy, Self-efficacy; HC= Health Counseling in-person; I= Intervention; IL= Illinois; IMB= Information-Motivation-Behavior model; IQR= Intra quantile range; LA= Los Angeles; M= Mean; LBW= Low Birth Weight; MA= Massachusetts; max. = maximum;; M= Mean; MET= Motivational Enhancement Therapy; MET-CBT= Motivational Enhancement Treatment – Cognitive Behavioural Therapy; MET-PS= Motivational Enhancement Therapy for Pregnant substance users; MI= Motivational Interview; mins= minutes; MIS= Mississippi; n/a = information not available; NC= North Carolina NM= New Mexico; NC= North Carolina; NICU= Neonatal Intensive Care Unit; NM= New Mexico; NS= non- significant; OC= Orange County; OH= Ohio; OR= Odds Ratio; P- value/ P= probability value; PA= Pennsylvania; PW= Pregnant Women; READS= establishing empathy, developing discrepancy, rolling with resistance, and supporting self-efficacy; RI= Rhodes Island; RR= Risk Ratio; RCT= Randomized Control Trials; SA= South Africa ; SAUP= Other-substance and alcohol use in pregnancy; SD= standard deviation ; SU= Substance Use; SUP= Substance use in Pregnancy; T= total participants; T-ACE= Tolerance, Annoyed, Cut Down, Eye-opener; TLFB= Alcohol Timeline Follow back; TWEAK= Tolerance Worry about drinking, Eye Opener, Amnesia, cut down on drinking; USA= United Sates of America; UK= United Kingdom; v/s = versus; wks.= weeks; yrs= years; 95% CI= 95% Confidence Interval*

**Table 2. National Institutes of Health (NIH) Quality Assessment Tool (24) for before-after (pre-post) studies with no control group**

| **Study** | **Criteria** | **Response** |
| --- | --- | --- |
| (25) de Vries et al, 2015 | 1. Was the study question or objective clearly stated? | Yes |
|  | 2. Were eligibility/selection criteria for the study population prespecified and clearly described? | Yes |
|  | 3. Were the participants in the study representative of those who would be eligible for the test/service/intervention in the general or clinical population of interest? | Yes |
|  | 4. Were all eligible participants that met the prespecified entry criteria enrolled? | Yes |
|  | 5. Was the sample size sufficiently large to provide confidence in the findings? | No |
|  | 6. Was the test/service/intervention clearly described and delivered consistently across the study population? | Yes |
|  | 7. Were the outcome measures prespecified, clearly defined, valid, reliable, and assessed consistently across all study participants? | Yes |
|  | 8. Were the people assessing the outcomes blinded to the participants' exposures/interventions? | Unknown |
|  | 9. Was the loss to follow-up after baseline 20% or less? Were those lost to follow-up accounted for in the analysis? | N/A (groups split based on completion of CM) |
|  | 10. Did the statistical methods examine changes in outcome measures from before to after the intervention? Were statistical tests done that provided p values for the pre-to-post changes? | Yes: AUDIT scores decrease significantly over time for women who completed case management (F = 23.323, p = 0.000). |
|  | 11. Were outcome measures of interest taken multiple times before the intervention and multiple times after the intervention (i.e., did they use an interrupted time-series design)? | Yes: 6 months, 12 months and 18 months |
|  | 12. If the intervention was conducted at a group level (e.g., a whole hospital, a community, etc.) did the statistical analysis take into account the use of individual-level data to determine effects at the group level? | Yes |

**Table 3. Quality assessment of cluster-randomized controlled trials (C-RCTs) using Cochrane tool (26)**

| Review | Study reference | Is it stated whether the reported C-RCT results are adjusted (i.e., in the text, tables, or forest plots)? | Is there a warning that CIs may be artificially narrow if unadjusted results are presented? | Are unadjusted results from C-RCTs excluded from meta-analysis? | Are unadjusted results adjusted using data presented in the trial reports? | If ICC was estimated, are sensitivity analyses carried out? | Are data from trials that adjust for clustering correctly extracted? | Are C-RCTs and RCTs grouped in the text, tables or forest plots (to investigate heterogeneity or to allow correct interpretation)? | Are C-RCTs grouped by unit of randomisation in the text, tables, or forest plots (to investigate heterogeneity or to allow correct interpretation)? |
| --- | --- | --- | --- | --- | --- | --- | --- | --- | --- |
| Alcohol use reduction/ neonatal outcomes (BI vs. control) | (27) Armstrong et al (2009) | Yes | No | No | Yes | N/A | Yes | No | No |
|  | (28) Van Der Wulp et al (2014) | No | No | No | No | Yes | Yes | No | No |
|  | (29) Marais (2011) | Yes | No | No | Yes | No | Yes | No | No |

References

1. Centre for Evidence Based Medicine. Randomised Controlled Trials (RCT) Critical Appraisal Sheet. Oxford University2001.

2. Chang G, McNamara TK, Orav EJ, Koby D, Lavigne A, Ludman B, et al. Brief intervention for prenatal alcohol use: a randomized trial. Obstet Gynecol. 2005;105(5 Pt 1):991-8.

3. Chang GW-H, Louise;Berman, Susan;Goetz, Margaret Ann. Brief intervention for alcohol use in pregnancy: A randomized trial. [References]: Addiction. Vol.94(10), 1999, pp. 1499-1508.; 1999.

4. Handmaker NS, Miller WR, Manicke M. Findings of a pilot study of motivational interviewing with pregnant drinkers. J Stud Alcohol. 1999;60(2):285-7.

5. O'Connor MJW, Shannon E. Brief intervention for alcohol use by pregnant women. [References]: American Journal of Public Health. Vol.97(2), 2007, pp. 252-258.; 2007.

6. Moura AAMD. Efeito do monitoramento por telefone de intervenções breves para uso de álcool e tabaco entre gestantes: Ensaio clínico randomizado: Universidade Federal De São Carlos

Centro De Ciências Biológicas E Da Saúde; 2019.

7. Ondersma SJ, Beatty JR, Svikis DS, Strickler RC, Tzilos GK, Chang G, et al. Computer-Delivered Screening and Brief Intervention for Alcohol Use in Pregnancy: A Pilot Randomized Trial. Alcohol Clin Exp Res. 2015;39(7):1219-26.

8. Osterman RL, Carle AC, Ammerman RT, Gates D. Single-session motivational intervention to decrease alcohol use during pregnancy. J Subst Abuse Treat. 2014;47(1):10-9.

9. Sheehan J, Gill A, Kelly BD. The effectiveness of a brief intervention to reduce alcohol consumption in pregnancy: a controlled trial. Ir J Psychol Med. 2014;31(3):175-89.

10. Tzilos GK, Sokol RJ, Ondersma SJ. A randomized phase I trial of a brief computer-delivered intervention for alcohol use during pregnancy. J Womens Health (Larchmt). 2011;20(10):1517-24.

11. Tzilos Wernette G, Plegue M, Kahler CW, Sen A, Zlotnick C. A Pilot Randomized Controlled Trial of a Computer-Delivered Brief Intervention for Substance Use and Risky Sex During Pregnancy. J Womens Health (Larchmt). 2018;27(1):83-92.

12. Rubio DM, Day NL, Conigliaro J, Hanusa BH, Larkby C, McNeil M, et al. Brief motivational enhancement intervention to prevent or reduce postpartum alcohol use: a single-blinded, randomized controlled effectiveness trial. J Subst Abuse Treat. 2014;46(3):382-9.

13. Peles E, Sason A, Bloch M, Maslovitz S, Dollberg S, Many A, et al. The Prevalence of Alcohol, Substance and Cigarettes Exposure among Pregnant Women within a General Hospital and the Compliance to Brief Intervention for Exposure Reduction. Isr J Psychiatry Relat Sci. 2014;51(4):248-56.

14. Osterman RL, Dyehouse J. Effects of a motivational interviewing intervention to decrease prenatal alcohol use. West J Nurs Res. 2012;34(4):434-54.

15. Reynolds KD, Coombs DW, Lowe JB, Peterson PL, Gayoso E. Evaluation of a Self-Help Program to Reduce Alcohol Consumption among Pregnant Women. International Journal of the Addictions. 1995;30(4):427-43.

16. Waterson EJ, Murray-Lyon I. Preventing fetal alcohol effects; a trial of three methods of giving information in the antenatal clinic. Health Education Research. 1990;5:53-61.

17. Nilsen P, Holmqvist M, Bendtsen P, Hultgren E, Cedergren M. Is questionnaire-based alcohol counseling more effective for pregnant women than standard maternity care? J Womens Health (Larchmt). 2010;19(1):161-7.

18. Sarvela PD, Ford TD. An evaluation of a substance abuse education program for Mississippi delta pregnant adolescents. J Sch Health. 1993;63(3):147-52.

19. Yonkers KA, Dailey JI, Gilstad-Hayden K, Ondersma SJ, Forray A, Olmstead TA, et al. Abstinence outcomes among women in reproductive health centers administered clinician or electronic brief interventions. J Subst Abuse Treat. 2020;113:107995.

20. Joya X, Mazarico E, Ramis J, Pacifici R, Salat-Batlle J, Mortali C, et al. Segmental hair analysis to assess effectiveness of single-session motivational intervention to stop ethanol use during pregnancy. Drug Alcohol Depend. 2016;158:45-51.

21. Yonkers KA, Forray A, Howell HB, Gotman N, Kershaw T, Rounsaville BJ, et al. Motivational enhancement therapy coupled with cognitive behavioral therapy versus brief advice: a randomized trial for treatment of hazardous substance use in pregnancy and after delivery. Gen Hosp Psychiatry. 2012;34(5):439-49.

22. Winhusen T, Kropp F, Babcock D, Hague D, Erickson SJ, Renz C, et al. Motivational enhancement therapy to improve treatment utilization and outcome in pregnant substance users. J Subst Abuse Treat. 2008;35(2):161-73.

23. Meberg A, Halvorsen B, Holter B, Ek IJ, Askeland A, Gaaserud W, et al. Moderate alcohol consumption—need for intervention programs in pregnancy? Acta Obstetricia et Gynecologica Scandinavica. 1986;65(8):861-4.

24. Ma L-L, Wang Y-Y, Yang Z-H, Huang D, Weng H, Zeng X-T. Methodological quality (risk of bias) assessment tools for primary and secondary medical studies: what are they and which is better? Military Medical Research. 2020;7(1):7.

25. de Vries MM, Joubert B, Cloete M, Roux S, Baca BA, Hasken JM, et al. Indicated Prevention of Fetal Alcohol Spectrum Disorders in South Africa: Effectiveness of Case Management. Int J Environ Res Public Health. 2015;13(1):ijerph13010076-ijerph.

26. Richardson M, Garner P, Donegan S. Cluster Randomised Trials in Cochrane Reviews: Evaluation of Methodological and Reporting Practice. PLoS One. 2016;11(3):e0151818.

27. Armstrong MA, Kaskutas LA, Witbrodt J, Taillac CJ, Hung Y-Y, Osejo VM, et al. Using drink size to talk about drinking during pregnancy: a randomized clinical trial of Early Start Plus. Soc Work Health Care. 2009;48(1):90-103.

28. van der Wulp NY, Hoving C, Eijmael K, Candel MJ, van Dalen W, De Vries H. Reducing alcohol use during pregnancy via health counseling by midwives and internet-based computer-tailored feedback: a cluster randomized trial. J Med Internet Res. 2014;16(12):e274.

29. Marais S, Jordaan E, Viljoen D, Olivier L, de Waal J, Poole C. The effect of brief interventions on the drinking behaviour of pregnant women in a high‐risk rural South African community: a cluster randomised trial. Early Child Development and Care. 2011;181(4):463-74.
