# Supplementary material for: Effectiveness of brief alcohol interventions for pregnant women: a systematic literature review and meta-analysis
Source: BMC Pregnancy Childbirth. 2023 Jan 24;23:61. doi: 10.1186/s12884-023-05344-8 (PMC9872314; doi:10.1186/s12884-023-05344-8)
Supplement: Supplementary file 6 — Additional file 6. Assessment in the certainty of evidence for each outcome included in the meta-analysis using the GRADE Approach. [file 12884_2023_5344_MOESM6_ESM.docx]

**Additional File 6. Assessment in the certainty of evidence for each outcome included in the meta-analysis using the GRADE Approach (1)**

| **Outcome description** | **Type of studies included in the meta-analyses** | **Number of participants in each condition (studies)** | **Statistical findings** | **Risk of bias** | **Consistency of effect** | **Imprecision** | **Indirectness** | **Certainty of the evidence** *(high, moderate, low, or very low)* |
| --- | --- | --- | --- | --- | --- | --- | --- | --- |
| Odds of alcohol abstinence during remainder of pregnancy (Fig 2) | RCTs + Quasi-experimental | 2,070 intervention; 1,561 control (10 studies) ^(2-11)^ | OR=1.86, 95% CI=1.15 – 2.13, moderate heterogeneity = 46.75%, p=0.00 | Moderate  Comments:  6 of 10 studies have methodological concerns with respect to randomization, blinding, participant follow-up/analysis and/or blinding/use of objective measures. | Consistent  Comments: 95% CIs of all included studies overlap and heterogeneity might not be important | Modest precision  Comments:  95% CI satisfactorily narrow; sample size large (OIS calculated as 859) and event rate satisfactory | Indirect  Comments: Studies included BI and control groups who were not comparable at baseline (e.g., alcohol use behaviour); outcome measurements and BI/control comparisons are direct in 6 studies. | Low |
|  | RCTs and C-RCT | 642 intervention; 698 control=1340 (8 studies) ^(2-5, 7, 8, 10, 11)^ | OR=1.86, 95% CI=1.39 – 2.49, heterogeneity might not be important= 16.76%, p=0.00 | Moderate  Comments:  8/8 studies randomized and had no selective reporting; 5/8 allocation concealment; 3/8: blinding of out outcome assessor or statistical analyst; 6/8: accounted for the loss to follow up and adhered to intention to treat analysis; 5/8: studies showed that the BI and control groups had comparable baseline characteristics. | Consistent  Comments: 95% CIs of all included studies over-lap and heterogeneity might not be important | Modest precision  Comments:  95% CI satisfactorily narrow; sample size large (OIS calculated as 225) and event rate satisfactory | Indirect  Comments: The population of interest across these studies differed in their baseline alcohol use, biological age; difference in the components of the intervention and control groups, and gestational age at which the intervention was conducted; outcome measurement is direct; intervention and control comparisons are direct (12) | Low |
| Reduction in mean AUDIT scores (Fig 3) | RCTs + Quasi-experimental | 308 intervention; 302 control (3 studies) ^(13-15)^ | Hedge’s g = 0.10, 95% CI= - 0.06 to 0.26, heterogeneity that can be ignored=0.0%, p=0.17 | High  Comments: 3 of 3 studies did not use blinding and/or objective measures. 2 of 3 studies used intervention and control groups who were not similar at baseline. | Consistent  Comments: 95% CIs of all included studies overlap and heterogeneity can be ignored | Low precision  Comments: Imprecision: The 95% CI includes 0, sample size is > 400 (as is recommended for continuous outcome measures) (12); not sure about the minimum difference one expects to detect | Direct  Comments: Studies included BI and control groups who were not comparable at baseline (e.g., alcohol use behaviour); outcome measurements and BI/control comparisons are direct | Low |
|  | RCTs | 294 intervention; 293 control=587 (2 studies) ^(13)^ | Hedge’s g = 0.07, 95% CI= - 0.09 to 0.23, heterogeneity might not be important =0.0%, p=0.87 | High  Comments:  Risk of bias: 2/2 studies were randomized; 1/2 allocation concealment; 0/2: blinding of out outcome assessor or statistical analyst; 2/2: accounted for the loss to follow up and adhered to intention to treat analysis, and 1/2 studies showed that the BI and control groups had comparable baseline characteristics | Consistent  Comments:  95% CIs of all included studies over-lap and heterogeneity might not be important | Low precision  Comments: Imprecision: The 95% CI includes 0, sample size is > 400 (as is recommended for continuous outcome measures) (12); not sure about the minimum difference one expects to detect | Direct  Comments:  The population of interest across these studies differed in their baseline alcohol use, biological age; difference in the components of the intervention and control groups, and gestational age at which the intervention was conducted; outcome measurement is direct; intervention and control comparisons are direct | Low |
| Reduction in mean drinks per week (Fig 4) | C-RCT | 58 intervention; 108 control (1 study with two arms) ^(5)^ | Cohen’s d= - 0.21, 95%CI = - 0.78 to 0.36, substantial heterogeneity =67.24%; p=0.47 | High  Comments: Risk of bias: was randomized; had allocation concealment; No blinding to outcome assessor or statistical analyst; did not account for loss-to follow-up; conducted intention to treat analysis; no selective reporting; although baseline characteristics for BI and controls differed- they were accounted for in the analysis (DD’s comment:  Findings based on one publication, in which the intervention and control groups were not comparable at baseline and not all participants were accounted for in the analysis) | Cannot ascertain (only 1 study) | Very low precision  Comments: Imprecision: The 95%CI includes 0, sample size is < 400; not sure about the minimum difference one expects to detect | Cannot ascertain (only one study) | Low |
| Increase in mean birthweight (Fig 6) | RCTs + Quasi-experimental | 191 intervention; 215 control (3 studies) ^(15-17)^ | Cohen’s d=0.16, 95%CI= -0.36 to 0.68, with considerable – substantial heterogeneity = 81.40%; p=0.54 | Moderate  Comments: Studies did not use blinding and/or objective measures. 1 of 3 studies included intervention and control groups who were not similar at baseline | Inconsistent  Comments: 95% CIs of all 3 included studies do not over-lap, and has substantial heterogeneity | Very low precision  Comments: The 95%CI includes 0, sample size is < 400; not sure about the minimum difference one expects to detect | Indirect  Comments: 2 of 3 studies included groups who were comparable at baseline; outcome measurement is direct; intervention and control comparisons are direct | Low |
|  | RCTs | 150 intervention; 149=299 control (2 studies) (16, 17) | Cohen’s d=0.131  (95%CI= -0.74, 1.01), with considerable – substantial heterogeneity = 87.43%; p=0.00 | Moderate  Comments:  2/2 studies randomized; 2/2: allocation concealment; 1/2: Binding to accessor; 2/2 accounted for loss to follow-up; ½ : conducted intention to treat; 2/2: accounted for baseline characteristics or adjusted for them when not comparable | Inconsistent  Comments: 95% CIs of all 2 included studies do not over-lap, and has substantial heterogeneity | Very low precision  Comments: The 95%CI includes 0, sample size is < 400; not sure about the minimum difference one expects to detect | Indirect  Comments: The population of interest across these studies differed in their baseline alcohol use, biological age; difference in the components of the intervention and control groups, and gestational age at which the intervention was conducted; outcome measurement is direct; intervention and control comparisons are direct | Low |
| Decreased odds of low birthweight (Fig 7) | RCT and C-RCT | 28 intervention; 33 control (2 studies) ^(11, 18)^ | OR=1.02, 95%CI=0.44 to 2.40, moderate heterogeneity=59.03%; p=0.96 | Moderate  Comments: 2/2 randomized; ½: Allocation concealment; 0/2: Blinded to assessor or statistical analyst; 1/2: accounted for lost to follow-up and conducted intention to treat analysis; 0/2: did not adjust for baseline characteristics  (DD: 2 of 2 studies have no or unknown comparability of treatment and control groups. One study also does not use randomization at the individual level (C-RCT), does not use blinding and/or objective measures, and does not account for all participants in the analysis.  ) | Inconsistent  Comments: The 95% CIs of all the 3 studies overlap, but the heterogeneity is moderate | Very low precision  Comments:  The 95%CI includes 1~~.~~ | Indirect  Comments: The population of interest across these studies differed in their baseline alcohol use, biological age; difference in the components of the intervention and control groups, and gestational age at which the intervention was conducted; outcome measurement is direct; intervention and control comparisons are direct | Low |
| Decreased odds of preterm birth (Fig 8) | RCT, C-RCT | 47 intervention; 79 control (2 studies) ^(11, 18)^ | OR=0.67, 95%CI= 0.46 to 0.98, small heterogeneity that can be ignored = 0.00%; p=0.04 | Moderate  Comments: 2/2 randomized; ½: Allocation concealment; 0/2: Blinded to assessor or statistical analyst; 1/2: accounted for lost to follow-up and conducted intention to treat analysis; 0/2: did not adjust for baseline characteristics (DD: 2 of 2 studies have no or unknown comparability of treatment and control groups and no or known blinding of participants and use of objective measures. One study also does not use randomization at the individual level (C-RCT) and does not account for all participants in the analysis.) | Consistent  Comments:  The 95%CIs of two studies are consistent, and the heterogeneity might not be important | Low precision  Comments: Although 95% CIs does not encompass 1 but it is close to no effect | Indirect  Comments: The population of interest across these studies differed in their baseline alcohol use, biological age; difference in the components of the intervention and control groups, and gestational age at which the intervention was conducted; outcome measurement is direct; intervention and control comparisons are direct | Low |

Publication bias not assessed since there were not enough studies.

References

1. Meader N, King K, Llewellyn A, Norman G, Brown J, Rodgers M, et al. A checklist designed to aid consistency and reproducibility of GRADE assessments: development and pilot validation. Syst Rev. 2014;3:82.

2. Ondersma SJ, Beatty JR, Svikis DS, Strickler RC, Tzilos GK, Chang G, et al. Computer-Delivered Screening and Brief Intervention for Alcohol Use in Pregnancy: A Pilot Randomized Trial. Alcohol Clin Exp Res. 2015;39(7):1219-26.

3. Chang GW-H, Louise;Berman, Susan;Goetz, Margaret Ann. Brief intervention for alcohol use in pregnancy: A randomized trial. [References]: Addiction. Vol.94(10), 1999, pp. 1499-1508.; 1999.

4. Handmaker NS, Miller WR, Manicke M. Findings of a pilot study of motivational interviewing with pregnant drinkers. J Stud Alcohol. 1999;60(2):285-7.

5. van der Wulp NY, Hoving C, Eijmael K, Candel MJ, van Dalen W, De Vries H. Reducing alcohol use during pregnancy via health counseling by midwives and internet-based computer-tailored feedback: a cluster randomized trial. J Med Internet Res. 2014;16(12):e274.

6. Meberg A, Halvorsen B, Holter B, Ek IJ, Askeland A, Gaaserud W, et al. Moderate alcohol consumption—need for intervention programs in pregnancy? Acta Obstetricia et Gynecologica Scandinavica. 1986;65(8):861-4.

7. Joya X, Mazarico E, Ramis J, Pacifici R, Salat-Batlle J, Mortali C, et al. Segmental hair analysis to assess effectiveness of single-session motivational intervention to stop ethanol use during pregnancy. Drug and Alcohol Dependence. 2016;158:45-51.

8. Reynolds KD, Coombs DW, Lowe JB, Peterson PL, Gayoso E. Evaluation of a Self-Help Program to Reduce Alcohol Consumption among Pregnant Women. International Journal of the Addictions. 1995;30(4):427-43.

9. Nilsen P, Holmqvist M, Bendtsen P, Hultgren E, Cedergren M. Is questionnaire-based alcohol counseling more effective for pregnant women than standard maternity care? J Womens Health (Larchmt). 2010;19(1):161-7.

10. Yonkers KA, Dailey JI, Gilstad-Hayden K, Ondersma SJ, Forray A, Olmstead TA, et al. Abstinence outcomes among women in reproductive health centers administered clinician or electronic brief interventions. J Subst Abuse Treat. 2020;113:107995.

11. Yonkers KA, Forray A, Howell HB, Gotman N, Kershaw T, Rounsaville BJ, et al. Motivational enhancement therapy coupled with cognitive behavioral therapy versus brief advice: a randomized trial for treatment of hazardous substance use in pregnancy and after delivery. Gen Hosp Psychiatry. 2012;34(5):439-49.

12. Guyatt GH, Oxman AD, Kunz R, Woodcock J, Brozek J, Helfand M, et al. GRADE guidelines: 8. Rating the quality of evidence&#x2014;indirectness. Journal of Clinical Epidemiology. 2011;64(12):1303-10.

13. Osterman RL, Carle AC, Ammerman RT, Gates D. Single-session motivational intervention to decrease alcohol use during pregnancy. J Subst Abuse Treat. 2014;47(1):10-9.

14. Sheehan J, Gill A, Kelly BD. The effectiveness of a brief intervention to reduce alcohol consumption in pregnancy: a controlled trial. Ir J Psychol Med. 2014;31(3):175-89.

15. Peles E, Sason A, Bloch M, Maslovitz S, Dollberg S, Many A, et al. The Prevalence of Alcohol, Substance and Cigarettes Exposure among Pregnant Women within a General Hospital and the Compliance to Brief Intervention for Exposure Reduction. Isr J Psychiatry Relat Sci. 2014;51(4):248-56.

16. Tzilos GK, Sokol RJ, Ondersma SJ. A randomized phase I trial of a brief computer-delivered intervention for alcohol use during pregnancy. J Womens Health (Larchmt). 2011;20(10):1517-24.

17. Rubio DM, Day NL, Conigliaro J, Hanusa BH, Larkby C, McNeil M, et al. Brief motivational enhancement intervention to prevent or reduce postpartum alcohol use: a single-blinded, randomized controlled effectiveness trial. J Subst Abuse Treat. 2014;46(3):382-9.

18. Armstrong MA, Kaskutas LA, Witbrodt J, Taillac CJ, Hung Y-Y, Osejo VM, et al. Using drink size to talk about drinking during pregnancy: a randomized clinical trial of Early Start Plus. Soc Work Health Care. 2009;48(1):90-103.
